# AP2X-1 is a negative regulator of *Toxoplasma gondii* sexual commitment

**DOI:** 10.1128/mbio.00052-25

**Published:** 2025-08-18

**Authors:** Li-Xiu Sun, Meng Wang, Tian-Yu Zhang, Hany M. Elsheikha, Zhi-Wei Zhang, Xiao-Nan Zheng, Bao-Quan Fu, Xing-Quan Zhu, Guo-Hua Liu, Jin-Lei Wang

**Affiliations:** 1 State Key Laboratory for Animal Disease Control and Prevention, Key Laboratory of Veterinary Parasitology of Gansu Province, Lanzhou Veterinary Research Institute, Chinese Academy of Agricultural Sciences 659819, Lanzhou, Gansu, People's Republic of China; 2 Research Center for Parasites & Vectors, College of Veterinary Medicine, Hunan Agricultural University 12575, Changsha, Hunan, People's Republic of China; 3 School of Veterinary Medicine and Science, Faculty of Medicine and Health Sciences, Sutton Bonington Campus, University of Nottingham 6123, Nottingham, United Kingdom; 4 Laboratory of Parasitic Diseases, College of Veterinary Medicine, Shanxi Agricultural University 74600, Taiyuan, Shanxi, People's Republic of China; UT Southwestern Medical Center, Dallas, Texas, USA

**Keywords:** *Toxoplasma gondii*, AP2X-1, HDAC3/MORC complex, gene expression, sexual development, transcription

## Abstract

*Toxoplasma gondii* is a widespread protozoan with a complex life cycle, characterized by transitions between various hosts and developmental stages, each tailored to a specific niche within its host. However, the regulatory mechanisms governing these life cycle transitions are not well understood. In this study, we investigated the AP2 factor AP2X-1, which is expressed during the tachyzoite and bradyzoite stages but decreases in the mature merozoite stage. Knockout of *ap2X-1* significantly impaired tachyzoite invasion and replication while increasing the frequency of bradyzoite differentiation. As a component associated with the HDAC3/MORC complex, loss of *ap2X-1* led to the upregulation of bradyzoite- and sexual stage-specific genes. Single-cell sequencing revealed that the *ap2X-1* knockout strain exhibited a mixed population of tachyzoite-, bradyzoite-, merozoite-, and sporozoite-like parasites. Cleavage under targets and tagmentation analysis revealed a substantial overlap between AP2X-1 and the HDAC3/MORC complex at the promoters of bradyzoite- and sexual stage-specific genes. Additionally, assay for transposase-accessible chromatin with high-throughput sequencing analysis demonstrated that AP2X-1 influences chromatin compaction and accessibility, suggesting that AP2X-1 may modulate the function of the HDAC3/MORC complex to facilitate the repression of bradyzoite differentiation and sexual commitment. Loss of *ap2X-1* resulted in significant attenuation of *T. gondii* virulence and decreased brain cyst formation *in vivo*. These findings identify AP2X-1 as a critical negative regulator of *T. gondii* sexual development.

**IMPORTANCE:**

*Toxoplasma gondii* undergoes a complex life cycle characterized by alternating developmental stages. The genetic reprogramming mechanisms driving these stage transitions remain largely unknown. In this study, we identified the AP2 factor AP2X-1 as a critical regulator important for *T. gondii* growth and life cycle progression. Our findings suggest that AP2X-1 functions as a repressor by modulating the function or influencing the association of the HDAC3/MORC complex at the promoters of bradyzoite- and sexual stage-specific genes, leading to chromatin compaction, restricting DNA accessibility and thereby repressing the transcription of genes required for bradyzoite formation and sexual commitment. Deletion of *ap2X-1* significantly reduced *T. gondii* virulence and its ability to form brain cysts. These findings reveal a previously unknown regulatory pathway controlling sexual development in *T. gondii*, providing new insights into its underlying mechanisms.

## INTRODUCTION

*Toxoplasma gondii* is an obligate intracellular protozoan capable of infecting a broad range of warm-blooded animals, with a complex life cycle that alternates between distinct developmental stages (1, 2). The parasite’s sexual cycle is confined to the feline intestinal epithelium, culminating in the formation and shedding of oocysts in the feces. In contrast, its asexual cycle occurs in a broad range of mammalian and avian intermediate hosts, involving the rapid replication of tachyzoites and the formation of bradyzoites (3, 4).

Tachyzoite, the fast-replicating form, can transition into quiescent bradyzoites under the pressure of the host’s immune response (5). Bradyzoite development is characterized by slowed growth and the formation of tissue cysts, which can persist for prolonged periods, potentially throughout the host’s entire lifespan (5, 6). Importantly, the host’s immune system is often unable to clear these tissue cysts effectively (7). The tissue cysts play a pivotal role in *T. gondii* pathogenesis and transmission (8). When felines ingest tissue cysts, the bradyzoites are released, invade intestinal epithelial cells, and undergo multiple rounds of schizogonic replication, producing merozoites (3, 4, 9). These pre-sexual stages subsequently undergo gametocytogenesis, leading to the formation of male microgametes and female macrogametes. After fertilization, immature oocysts are excreted in the feces (3, 4, 9). Upon exposure to air, these oocysts sporulate and become infectious, enabling transmission (3, 4). Despite the critical role oocysts play in the parasite’s biology and epidemiology, the molecular mechanisms underlying the stage conversion between the various life cycle forms remain incompletely understood. A deeper understanding of these molecular mechanisms is crucial for controlling both the transmission and pathogenesis of *T. gondii*.

As one of the most successful parasites, *T. gondii* has developed a highly dynamic and precise genetic reprogramming system to regulate stage conversion (10). A wide array of mRNAs and proteins is expressed in a stage-specific manner, enabling the parasite to adapt to various hosts and environmental niches throughout its life cycle (11). Among these regulatory proteins, the Apetala-2 (AP2) family of transcription factors plays a significant role. AP2 proteins are DNA-binding factors found in apicomplexan parasites (12), with at least 68 predicted AP2-containing proteins in the *T. gondii* genome (13). Among them, 24 AP2 factors are regulated during the tachyzoite cell cycle, 11 are induced under bradyzoite conditions, 27 are constitutively expressed, and 6 are not expressed during intermediate life cycle stages (14). In *T. gondii*, AP2 factors are integral to stage conversion regulation. For example, AP2XI-4 (15) and AP2IV-3 (16) act as transcriptional activators, while AP2IX-9 (16) and AP2IV-4 (17) serve as transcriptional repressors that regulate bradyzoite gene expression during tachyzoite-to-bradyzoite conversion. Several AP2 factors are also linked to epigenetic regulators, such as the GCN5b/ADA2a and HDAC3/MORC complexes, which help coordinate transcriptional regulation (11, 18).

Recent studies have identified at least 12 AP2 factors associated with the HDAC3/MORC complex, which plays a critical role in preventing the inappropriate expression of genes restricted to the sexual stages in felines (11, 19–21). For example, AP2XII-2 recruits the HDAC3/MORC complex to specific gene loci to repress sexual stage-specific genes, although it does not fully suppress sexual commitment. The loss of AP2XII-2 only dysregulates a small subset of mRNAs in *T. gondii* (21). Additionally, AP2XII-1 and AP2XI-2 form heterodimers that bind to merozoite promoters, recruiting the HDAC3/MORC complex to restrict chromatin accessibility and transcription during the tachyzoite stage. These factors are not expressed during the merozoite stage, allowing merozoite differentiation to proceed (20, 22, 23). Despite these insights, the specific roles of other AP2 factors associated with the HDAC3/MORC complex remain poorly understood and warrant further investigation.

In the present study, we focused on the function of AP2X-1, one of the 12 AP2 factors associated with the HDAC3/MORC complex. Genetic ablation of AP2X-1 had a significant impact on tachyzoite growth, with the knockout strain showing an increased capacity for tissue cyst formation *in vitro*. Comprehensive analyses, including transcriptome profiling, single-cell RNA sequencing, cleavage under targets and tagmentation (CUT&Tag), and assay for transposase-accessible chromatin with high-throughput sequencing (ATAC-seq), suggest that AP2X-1 influences the association of the HDAC3/MORC complex with the promoters of bradyzoite- and sexual stage-specific genes. This association appears to affect chromatin compaction and accessibility, contributing to the transcriptional repression of bradyzoite differentiation and commitment to sexual development.

## RESULTS

### The nuclear factor AP2X-1 is constitutively expressed in tachyzoites and bradyzoites but decreases in mature merozoites

The *ap2X-1* gene (TGME49_227900) is predicted to contain a single exon encoding a 120-kDa protein with 1,167 amino acids, though no definitive AP2 domain was identified through InterPro database predictions (https://www.ebi.ac.uk/interpro/). To further explore AP2X-1 protein expression, we engineered Pru parasites to express native AP2X-1 fused with a C-terminal 6HA epitope tag using CRISPR-Cas9-mediated homologous recombination. In the resulting Pru::AP2X-1-6HA strain (referred to as AP2X-1-6HA), successful tagging of AP2X-1 with 6HA was confirmed by PCR and DNA sequencing (Fig. S1A).

**Fig 1:**
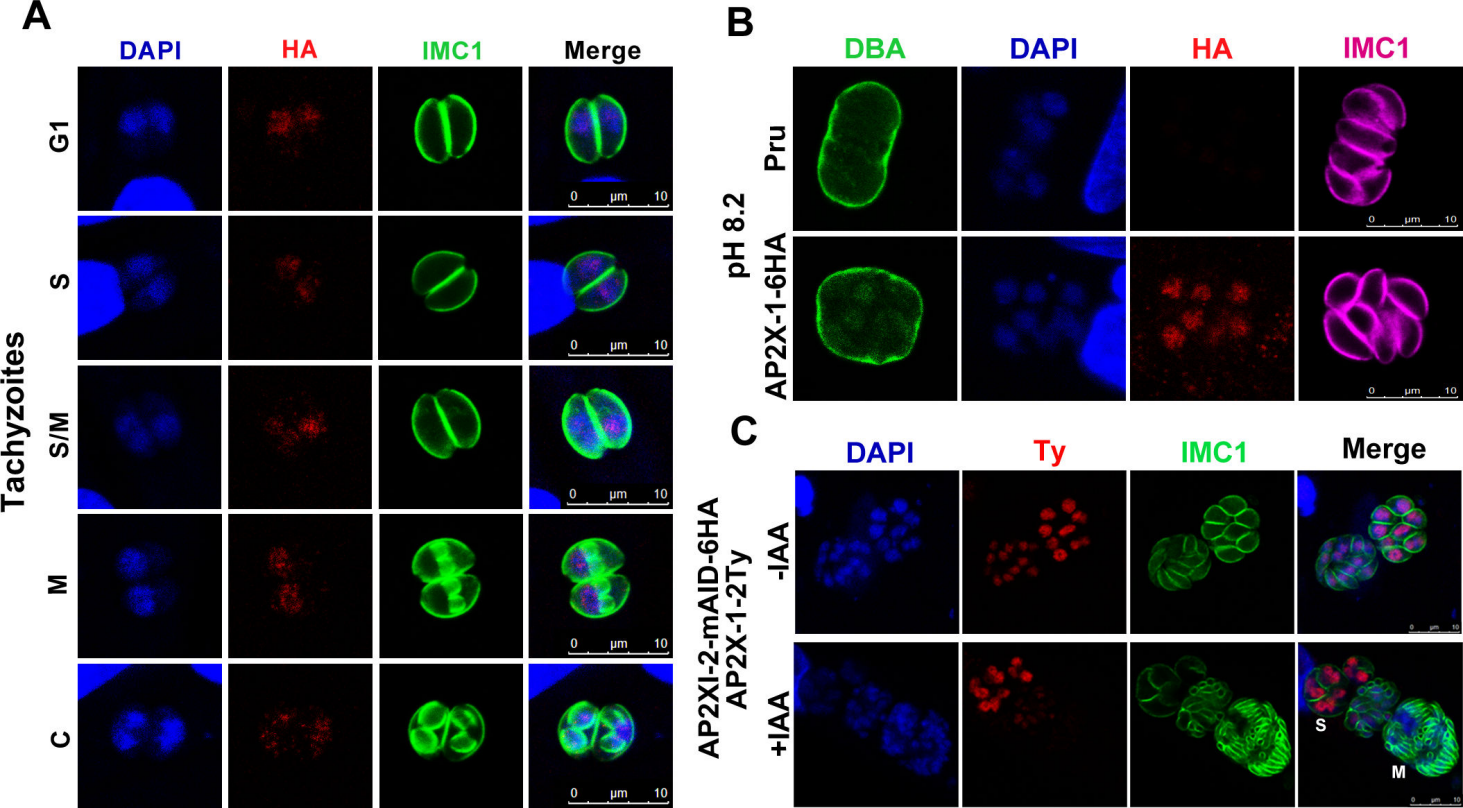
Subcellular localization of the nuclear factor AP2X-1 in *Toxoplasma gondii*. (**A**) AP2X-1 was consistently expressed throughout the cell cycle of *T. gondii* tachyzoites. IMC1 (green) marks the tachyzoite cell cycle. G1, gap phase; S, synthesis phase; M, mitotic phase; and C, cytokinesis. The expression of AP2X-1-6HA was detected using an anti-HA antibody (red), while DNA was visualized with 4′,6-diamidino-2-phenylindole (DAPI) (blue). Scale bar, 10 µm. (**B**) Subcellular localization of AP2X-1 in *T. gondii* bradyzoites induced by alkaline culture conditions for 2 days. The cyst wall was stained with FITC-labeled *Dolichos biflorus agglutinin* (DBA) (green), parasites were stained with IMC1 (magenta), AP2X-1-6HA was detected with anti-HA antibody (red), and DNA was visualized with DAPI (blue). Scale bar, 10 µm. (**C**) Subcellular localization of AP2X-1 during merogony induced by alkaline culture conditions for 3 days in the Pru-AP2XI-2-mAID-6HA strain. The parasites were stained with IMC1 (green), AP2X-1-2Ty was detected with anti-Ty antibody (red), and genomic DNA was visualized with DAPI (blue). M, merozoite and S, schizont. Scale bar, 10 µm.

Western blotting of tachyzoite lysate revealed a band corresponding to the expected size of AP2X-1 (Fig. S1B), with the presence of an additional band possibly indicating internal processing or degradation. An immunofluorescence assay was performed to assess subcellular localization and expression of AP2X-1 throughout the tachyzoite cell cycle, using the daughter cell budding marker IMC1 to identify the cell cycle stage. Results showed clear nuclear localization of AP2X-1 in tachyzoites, with consistent expression throughout the cell cycle (Fig. 1A). It is worth mentioning that a similar AP2X-1 expression pattern was observed in bradyzoites induced by alkaline medium treatment for 2 days (Fig. 1B). To examine the localization of AP2X-1 during merogony, we fused a 2Ty epitope tag to the endogenous C-terminus of AP2X-1 in the Pru-AP2XI-2-mAID-6HA strain, which serves as a model for studying *T. gondii* merogony *in vitro* (22). Immunofluorescence assay (IFA) revealed the nuclear localization of AP2X-1 in schizonts, while its presence decreased or was absent in mature merozoites (Fig. 1C). These results indicate that AP2X-1 is constitutively expressed in the tachyzoite and bradyzoite stages but decreases in the mature merozoite stage.

### AP2X-1 is critical for tachyzoite growth *in vitro*

**Fig 2:**
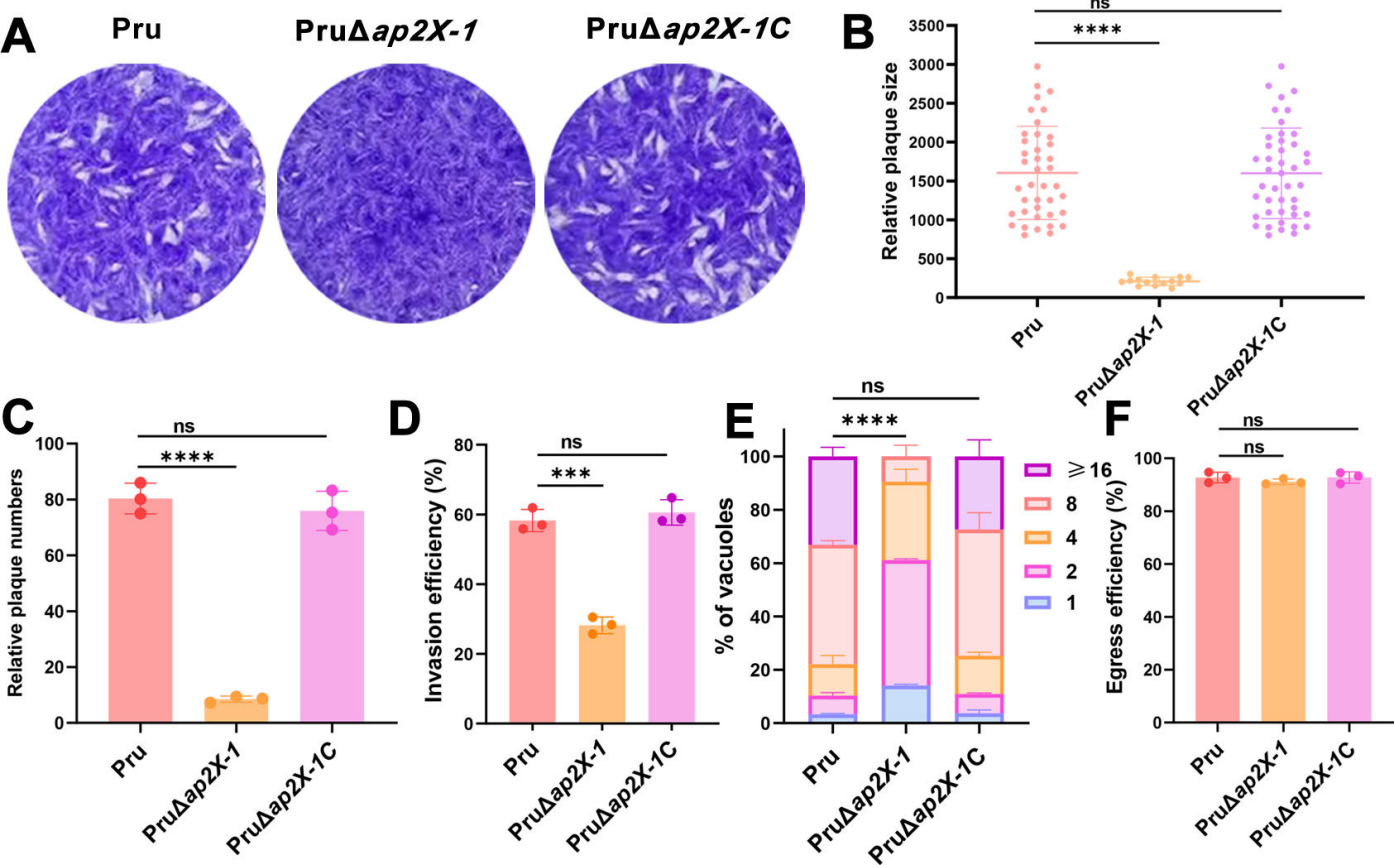
AP2X-1 is critical for the growth of *T. gondii in vitro*. (**A**) Representative images of plaques formed by Pru, PruΔ*ap2X-1,* and PruΔ*ap2X-1*C strains grown under normal culture conditions for 9 days. (**B**) The relative size of the plaques detected in panel **A**. Data represent the mean ± SD for three independent experiments. Statistical analysis was performed by a two-tailed, unpaired *t*-test, ^****^P < 0.0001. (**C**) The number of plaques detected in panel **A**. Data represent the mean ± SD for three independent experiments. Statistical analysis was performed by a two-tailed, unpaired *t*-test, ^****^P < 0.0001. (**D**) Invasion assay of Pru, PruΔ*ap2X-1,* and PruΔ*ap2X-1*C strains grown in human foreskin fibroblasts (HFFs). Data represent the mean ± SD for three independent experiments. Statistical analysis was performed by a two-tailed, unpaired *t*-test, ^***^P < 0.001. (**E**) Intracellular replication assay of Pru, PruΔ*ap2X-1,* and PruΔ*ap2X-1*C strains grown in HFFs for 36 h. At least 100 parasitophorous vacuoles (PVs) of each strain were examined in the experiment. Data represent the mean ± SD for three independent experiments. Statistical analysis was performed by two-way ANOVA with Tukey tests for multiple comparisons, ^****^P < 0.0001. (**F**) Egress assay of Pru, PruΔ*ap2X-1,* and PruΔ*ap2X-1*C strains. At least 100 PVs of each strain were examined in the experiment. Data represent the mean ± SD for three independent experiments. Statistical analysis was performed using a two-tailed, unpaired *t*-test. ns, not significant; *P* > 0.05.

To investigate its biological role, we disrupted the *ap2X-1* in the Pru strain, using CRISPR-Cas9-mediated homologous recombination. Following clonal isolation, the fragments of the AP2X-1 coding sequence were not amplified in the *ap2X-1* knockout strains but were successfully amplified in the wild-type Pru strain (~650 bp) by using diagnostic PCR4. Additional verification through PCR3 and PCR5 showed amplification of approximately 1,700-bp fragments in the mutant strains, absent in the parental Pru strain (Fig. S1C), confirming the successful deletion of *ap2X-1*. To assess the impact of *ap2X-1* deletion on the parasite infectivity, a standard plaque assay was performed, simulating the phases of *T. gondii’*s lytic cycles, including invasion, replication, and egress. After 9 days of infection, the PruΔ*ap2X-1* strain formed significantly smaller and fewer plaques compared to the wild-type Pru strain, indicating a marked reduction in the growth capacity (Fig. 2A through C).

Next, we examined the specific phases of the lytic cycle affected by *ap2X-1* deletion. First, we assessed invasion efficiency by calculating the ratio of intracellular tachyzoites to the total number of tachyzoites after 1 h of invasion. The results showed a 30% reduction in invasion efficiency in the PruΔ*ap2X-1* strain compared to the wild-type Pru strain (Fig. 2D). We then investigated parasite replication by counting the number of tachyzoites within parasitophorous vacuoles (PVs) after 36 h of infection. A significant reduction in replication efficiency was observed in the PruΔ*ap2X-1* strain (Fig. 2E). Finally, we evaluated parasite egress using a Ca^2+^ ionophore-induced calcium egress assay, which showed no significant difference in egress capacity between the PruΔ*ap2X-1* strain and the wild-type Pru strain (Fig. 2F). These findings suggest that the deletion of *ap2X-1* primarily affects the invasion and intracellular replication phases of *T. gondii*'s life cycle, leading to a reduction in *in vitro* growth.

To confirm that the altered phenotype was specifically due to the loss of *ap2X-1*, we complemented the PruΔ*ap2X-1* strain by introducing a C-terminal 2Ty-tagged wild-type copy of *ap2X-1*, driven by its native promoter, into the *hxgprt* locus. The successfully constructed complemented strain, PruΔ*ap2X-1*C, was verified by PCRs (Fig. S1D), immunofluorescence assay (Fig. S1E), and western blotting (Fig. S1E). Phenotypic analyses confirmed that complementation of AP2X-1 successfully restored the AP2X*-*1 function (Fig. 2A through F).

Given the crucial role of AP2X-1 in parasite growth, we further validated its function using the auxin-inducible degron (AID) system. In this system, the endogenous AP2X-1 was tagged with 3-indoleacetic acid (IAA)-inducible degradation domain (mAID-HA) at the C-terminus in the auxin receptor (TIR1) expressing the Pru-TIR1 strain (Fig. S2A). By fusing mAID-HA tag to AP2X-1 (referred to as AP2X-1-mAID), we were able to efficiently degrade the AP2X-1 protein following IAA treatment, as demonstrated by immunofluorescence and western blotting analyses (Fig. S2B and C). The resulting growth characteristics in the AP2X-1-depleted parasites mirrored those observed in the knockout strain (Fig. S2D through I).

### AP2X-1 associates with the HDAC3/MORC complex and modestly restricts chromatin accessibility

Recent studies have demonstrated that AP2X-1 is one of 12 AP2 factors that associate with the HDAC3/MORC complex, which suppresses felid-specific developmental genes by promoting heterochromatin formation (11, 19–21). To further explore the association of AP2X-1 with the HDAC3/MORC complex, we generated the AP2X-1-2Ty, MORC-2Ty, and HDAC3-2Ty strains. These strains were immunoprecipitated using anti-Ty-conjugated magnetic beads, followed by liquid chromatography-mass spectrometry (LC-MS) analysis. LC-MS analysis of the MORC-2Ty strain from three independent experiments revealed that MORC associates with AP2X-1 and HDAC3. Similarly, LC-MS analysis of the HDAC3-2Ty strain showed that HDAC3 associates with AP2X-1 and MORC. However, in the AP2X-1-2Ty strain, although HDAC3 peptide fragments were not detected, MORC peptide fragments were identified. This suggests that AP2X-1 may associate with HDAC3 indirectly, or the lack of HDAC3 detection may be due to some degree of AP2X-1 degradation during the process. Taken together, these data suggest that AP2X-1 associates with the HDAC3/MORC complex to exert its function. In addition, the hypothetical proteins TGME49_234230, TGME49_297880, TGME49_288720, and TGME49_262620 are co-associated with AP2X-1 and HDAC3/MORC complex (Data set S1).

**Fig 3:**
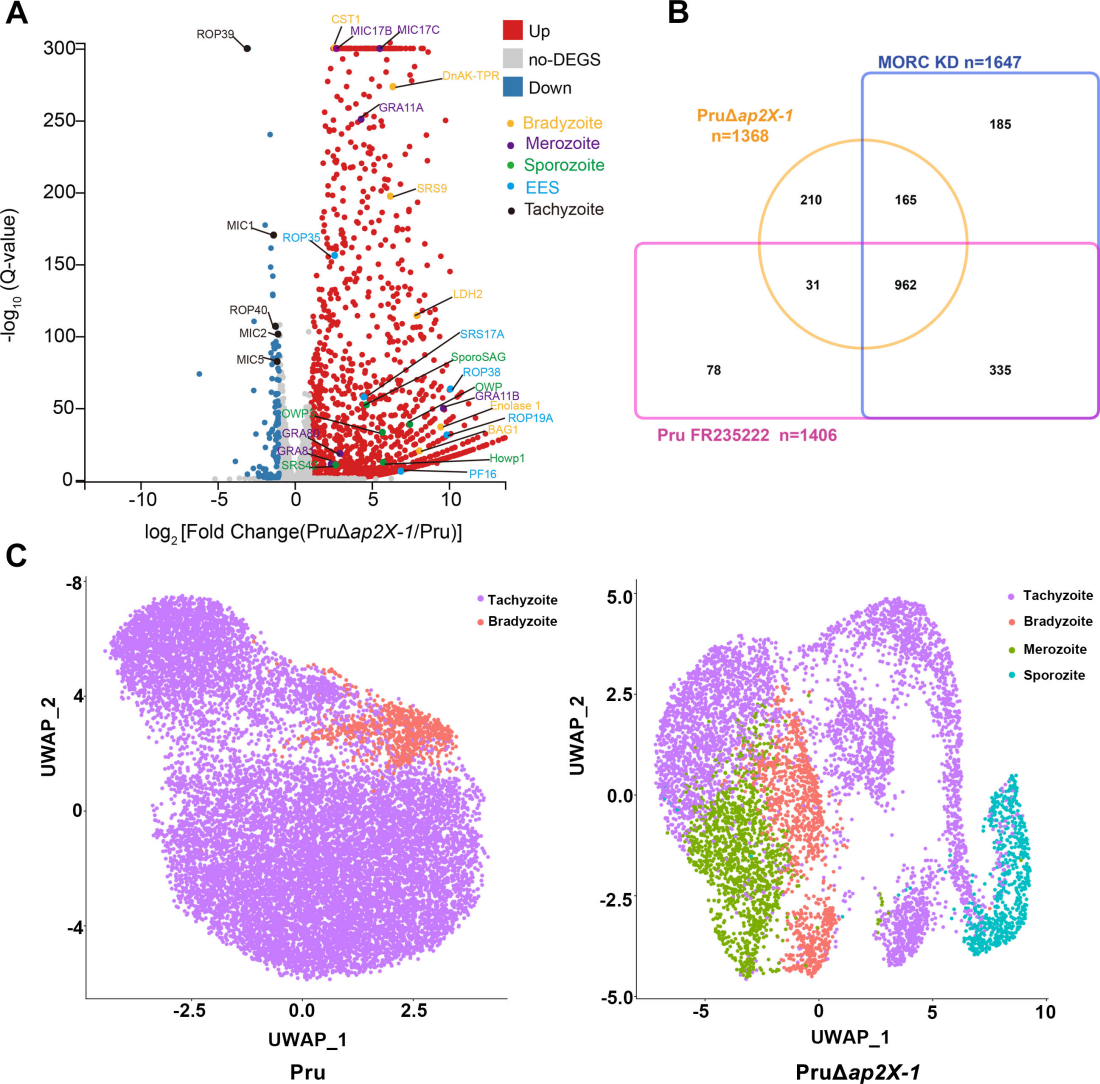
Knockout of *ap2X-1* induces transcription of bradyzoite and sexual stage-specific genes. (**A**) Volcano plot analysis of dysregulated genes in Pru and PruΔ*ap2X-1* strains. Upregulated genes are represented by red dots, downregulated genes by blue dots, and non-differentially regulated genes by gray dots. Genes highly expressed in merozoite, bradyzoite, sporozoite, and tachyzoite were analyzed from Data set S2. Data from three biological replicates were plotted, with a fold change of ≥2.0 or ≤−2.0 and *Q* value < 0.05 considered statistically significant. (**B**) Comparison of upregulated mRNAs after knockout of the *ap2X-1*, depletion of MORC, and FR235222-induced inhibition of HDAC3. (**C**) Clustering of tachyzoite-, bradyzoite-, merozoite-, and sporozoite-like genes in Pru and PruΔ*ap2X-1* strains, visualized using uniform manifold approximation and projection (UMAP) of single-cell transcriptomes. Dots are colored according to different clusters of *T. gondii*.

To further investigate the roles of *ap2X-1* in gene regulation, RNA sequencing was conducted to assess changes in gene expression following the deletion or depletion of *ap2X-1*. Genes with a fold change of ≥2.0 or ≤ −2.0 and *Q* value of <0.05 were deemed statistically significant in three biological replicates. Transcriptomic data revealed that *ap2X-1* deletion resulted in the altered expression of 1,473 genes (Fig. 3A). Of these, 1,368 genes were significantly upregulated (fold change of ≥2.0 and *Q* < 0.05), while 105 genes were significantly downregulated (fold change of ≤−2.0 and *Q* < 0.05), excluding *ap2X-1* itself (Data set S2). Interestingly, the majority of upregulated genes overlapped with those affected by auxin-induced depletion of MORC (82.38%, 1,127 out of 1,368) and HDAC3 inhibition (72.59%, 993 out of 1,368) (Fig. 3B; Data set S2). Similarly, RNA sequencing of AP2X-1-mAID-HA strain cultured under normal conditions with or without IAA revealed expression of 1,207 genes, with 1,195 significantly upregulated (fold change ≥ 2.0 and *Q* < 0.05) (Fig. S3A; Data set S3). In this case, 73.64% of the upregulated genes overlapped with MORC depletion (880 out of 1,195) and 66.69% with HDAC3 inhibition (797 out of 1,195) (Fig. S3B; Data set S3). The less pronounced transcriptomic changes upon depletion of AP2X-1, compared to its complete knockout, may result from residual AP2X-1 expression due to leakage. Furthermore, single-cell sequencing analysis revealed that the AP2X-1 knockout strain contained a mixed population of tachyzoite-, bradyzoite-, merozoite-, and sporozoite-like parasites, similar to the MORC-depleted strain (Fig. 3C). In contrast, the wild-type Pru strain exhibited a mixed population of tachyzoite- and bradyzoite-like parasites.

**Fig 4:**
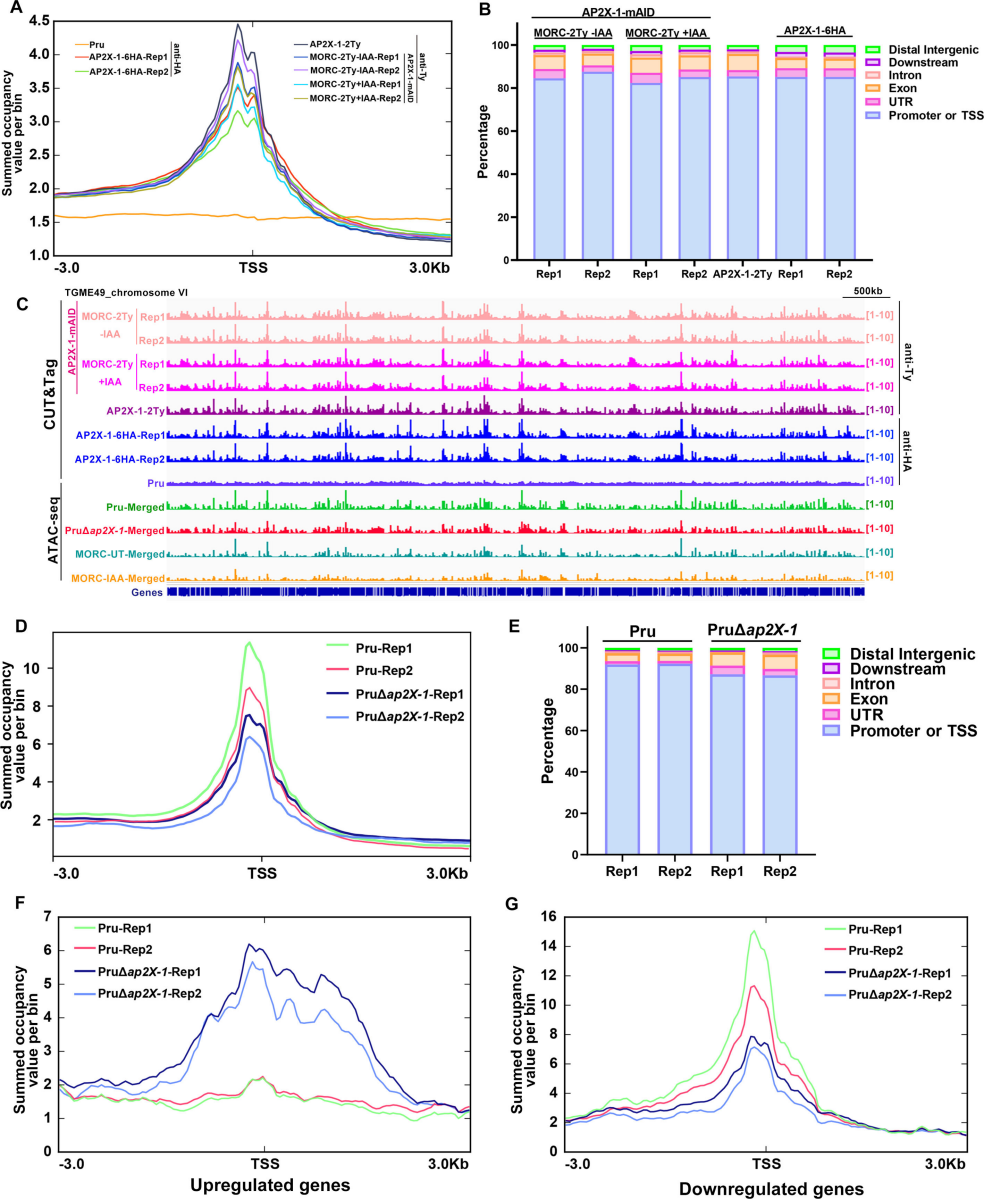
Loss of AP2X-1 affects chromatin association of the HDAC3/MORC complex. (**A**) Superposed profile plots indicate that AP2X-1 and MORC are associated with the transcriptional start sites of the genes. The AP2X-1-mAID-MORC-2Ty and AP2X-1-2Ty strains were incubated with mouse anti-Ty antibody, and the AP2X-1-6HA strain was incubated with mouse anti-HA antibody, which were used to assess the genomic loci of AP2X-1 or MORC. The AP2X-1-mAID-MORC-2Ty parasites were treated with or without IAA for 48 h for two biological replicates. The average signal profiles of each gene were plotted across a −3 to 3 kb region surrounding the TSS of the nearest *T. gondii* gene, with the average tag count of the enrichment shown on the *y*-axis. (**B**) Global distribution of significant peaks within genomic features for AP2X-1 or MORC CUT&Tag experiments. (**C**) Integrated Genome Viewer screenshots illustrate AP2X-1 and MORC enrichment across chromosome VI and chromatin accessibility (ATAC-seq) in Pru, PruΔ*ap2X-1,* and the MORC depletion strains, with read density shown on the *y*-axis. The AP2X-1-mAID-MORC-2Ty (+IAA/−IAA) and AP2X-1-2Ty strains were incubated with mouse anti-Ty antibody, and the AP2X-1-6HA strain was incubated with mouse anti-HA antibody. ATAC-seq data from two biological replicates were combined into a single data set for both the Pru and PruΔ*ap2X-1* strains. (**D**) Profile of summed occupancy shows chromatin accessibility (ATAC-seq) for the genes centered at the TSS (±3 kb) in Pru and PruΔ*ap2X-1* strains with the average tag count of the enrichment on the *y*-axis. The overlapped peaks in the two biological replicates were identified for the Pru and PruΔ*ap2X-1* strains. (**E**) Global distribution of significant peaks within genomic features for Pru and PruΔ*ap2X-1* strains based on ATAC-seq experiments. (**F and G**) The chromatin accessibility plot predicts nucleosomal occupancy. Mean occupancy profiles of ATAC-seq signals across upregulated genes (**F**) and downregulated genes (**G**) show enrichment at the TSS and phased mononucleosome fragments in the surrounding regions of Pru and PruΔ*ap2X-1* strains.

To explore how AP2X-1 silences gene expression, CUT&Tag analysis was performed on the AP2X-1-6HA, AP2X-1-2Ty, and AP2X-1-mAID-MORC-2Ty strains. The results displayed that AP2X-1-associated regions are widespread across the parasite genome (Fig. 4A and C; Data set S4). In the binding sites of AP2X-1, approximately 85.35% of peaks were located at the promoter regions or transcriptional start sites (TSSs) of the protein-coding genes (Fig. 4B; Data set S4). In addition, CUT&Tag showed that AP2X-1 significantly co-localized with MORC, with over 66.01% of AP2X-1-enriched regions overlapping with MORC-enriched peaks (Fig. 4A and C; Data set S4). Furthermore, gene expression analysis revealed that 48.03% of upregulated genes (657 out of 1,368) in the AP2X-1 depletion mutant were directly targeted by AP2X-1.

Given the critical role of the HDAC3/MORC complex in chromatin compaction and accessibility, we performed ATAC-seq on PruΔ*ap2X-1* and wild-type Pru strains to assess AP2X-1’s impact on chromatin regulation. ATAC-seq is a robust and streamlined method for profiling chromatin accessibility (24). Genome-wide analysis showed a slight decrease in chromatin accessibility in the PruΔ*ap2X-1* strain compared to the wild-type Pru strain (Fig. 4C and D; Data set S5), with approximately 86.97% of peaks located at promoter regions and TSS (Fig. 4E). ATAC-seq data for prominently upregulated and downregulated genes in PruΔ*ap2X-1* revealed modest changes in chromatin occupancy, consistent with the expected increase or decrease in accessibility for induced or repressed gene clusters, respectively (Fig. 4F and G).

These findings suggest that AP2X-1 may influence the function of the HDAC3/MORC complex to repress gene expression and limit chromatin accessibility at genes involved in bradyzoite differentiation and sexual development.

### Loss of *ap2X-1* induces bradyzoite differentiation

**Fig 5:**
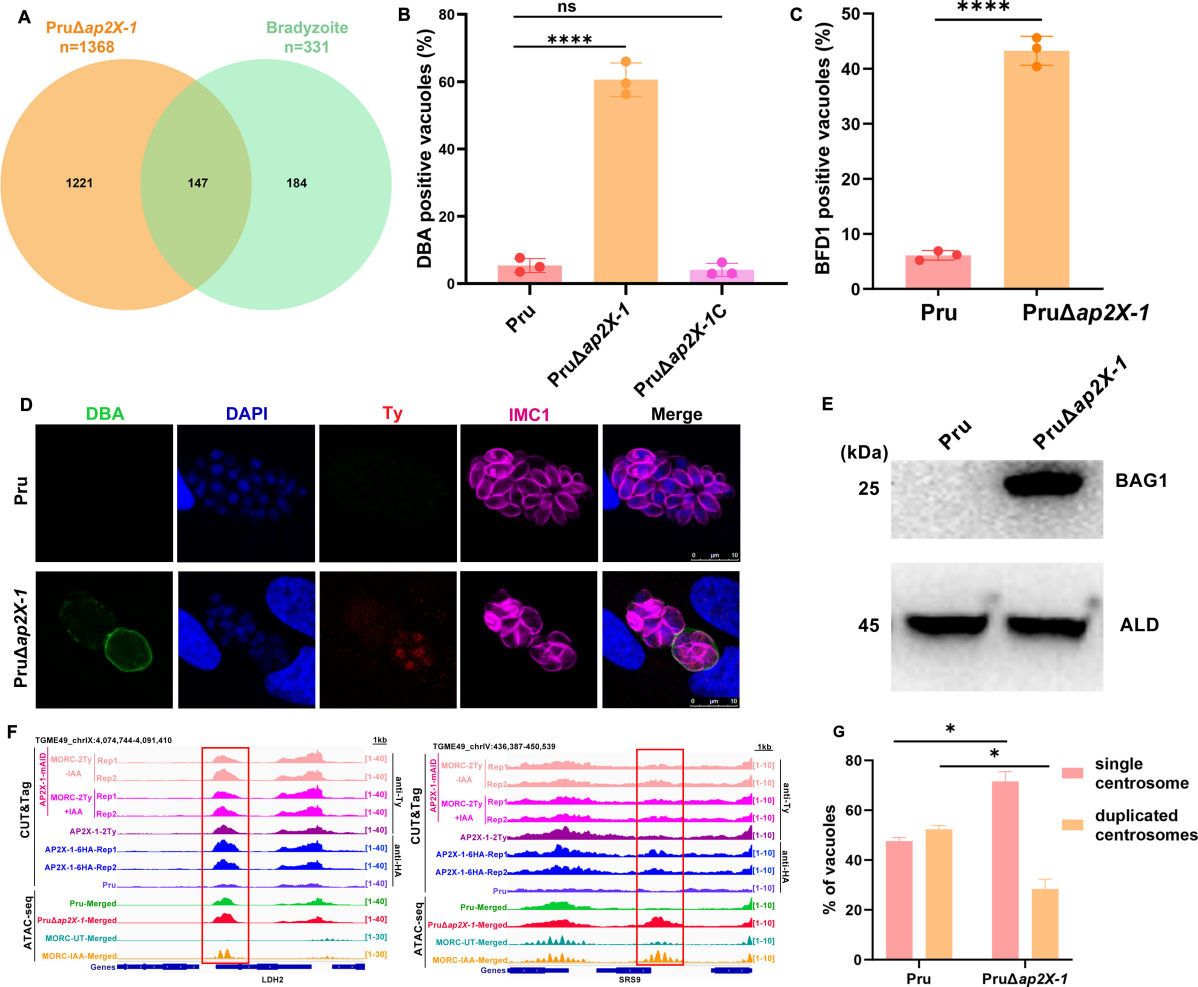
Knockout of *ap2X-1* induces the tachyzoite-to-bradyzoite conversion under normal culture conditions. (**A**) Venn diagram illustrates that disruption of *ap2X-1* induces the expression of bradyzoite-associated genes. Data for bradyzoite-associated genes were obtained from ToxoDB. (**B**) Knockout of *ap2X-1* increased spontaneous conversion from tachyzoites to bradyzoites under normal culture conditions. The mean ± SD was calculated from three biological replicates, with the percentage of DBA-positive PVs calculated from at least 100 PVs per replicate. Statistical analysis was performed using a two-tailed, unpaired *t*-test, ^****^P < 0.0001. (**C**) The graph displays the percentage of BFD1-positive vacuoles in Pru and PruΔ*ap2X-1* strains cultured in normal medium. The mean ± SD was calculated from three biological replicates, with the percentage of BFD1-positive PVs determined from at least 100 PVs per replicate. Statistical analysis was performed using a two-tailed, unpaired *t*-test, ^****^P< 0.0001. (**D**) IFA shows the bradyzoite protein expression of BFD1 in the PruΔ*ap2X-1* strain after 48 h. BFD1 was detected by a Ty antibody in PruΔ*ap2X-1*/BFD1-2Ty transgenic strain. Colors indicate magenta, anti-IMC1; green, DBA; red, anti-Ty; and blue, DAPI. Scale bar, 10 µm. (**E**) Western blotting confirms the expression of BAG1 in the PruΔ*ap2X-1* strain. Parasite lysates were incubated with anti-BAG, while anti-aldolase (ALD) was used as the loading control. (**F**) Integrated Genome Viewer screenshots of representative bradyzoite-associated genes (*LDH2* and *SRS9*) highlight CUT&Tag signal occupancy for AP2X-1-mAID-MORC-2Ty strain under untreated and post-depletion conditions, as well as AP2X-1-2Ty and AP2X-1-6HA strains under normal culture conditions. The AP2X-1-mAID-MORC-2Ty and AP2X-1-2Ty strains were incubated with mouse anti-Ty antibody, and the AP2X-1-6HA strain was incubated with mouse anti-HA antibody. Chromatin accessibility (ATAC-seq) profiles of the indicated strains are shown. ATAC-seq data from two biological replicates were combined into a single data set for both the Pru and PruΔ*ap2X-1* strains. (**G**) The percentage of centrosomes in Pru and PruΔ*ap2X-1* strain cultures in normal medium is shown. Data represent the mean ± SD from three biological replicates, with the percentage of centrosome-containing-PVs calculated from at least 100 PVs per replicate. Statistical analysis was performed by a two-tailed, unpaired *t*-test, ^****^P < 0.0001.

Transcriptomic data showed that a larger number of bradyzoite-associated genes (147/331), including *BAG1*, *LDH2,* and *CST1*, were significantly upregulated in the PruΔ*ap2X-1* strain cultured in normal medium (Fig. 5A; Fig. S3C). To verify whether the loss of *ap2X-1* triggers a tachyzoite-to-bradyzoite transition, human foreskin fibroblasts (HFFs) were infected with wild-type Pru and PruΔ*ap2X-1* strains under normal medium for 2 days, and the bradyzoite transformation rate was determined. The results demonstrated that approximately 59.08% of PruΔ*ap2X-1* PVs were positive for the bradyzoite cyst wall marker *Dolichos biflorus agglutinin* (DBA), significantly higher than the 5.37% observed in the wild-type Pru strain (Fig. 5B; Fig. S3D). Similar findings were observed in the auxin-induced depletion of AP2X-1-mAID-HA strain (Fig. S3E). Importantly, no noticeable changes in the morphology of the formed cysts were observed.

As expected, the bradyzoite master regulator BFD1 was expressed in approximately 43.25% of the PruΔ*ap2X-1* strain maintained in normal medium for 2 days (Fig. 5C and D). Additionally, the bradyzoite-specific protein BAG1 was detected on the PruΔ*ap2X-1* strain by western blotting (Fig. 5E). CUT&Tag analysis demonstrated that AP2X-1 and MORC were enriched at the promoter regions of some bradyzoite-specific genes, such as *LDH2*. Furthermore, ATAC-seq revealed that AP2X-1 knockout led to modestly increased chromatin accessibility, promoting the transcriptional activation of these genes—an effect similar to that observed in the MORC knockdown strain (Fig. 5F) (25).

Previous research has shown that developing bradyzoites undergo progressively extended G1 phase over successive rounds of cell division, ranging from hours to days or even weeks, and mature bradyzoites can remain in the G1 phase almost indefinitely, rarely dividing (26–28). To examine the cell cycle stages of the PruΔ*ap2X-1* strain, IFAs were performed using markers for the centrosome (Centrin-1) and daughter cell budding (IMC1). In a typical asynchronous population of the Pru tachyzoites, approximately 50% are in the G1 phase (single centrosome), while the other 50% are in the S, M, and C phases, characterized by DNA replication (duplicated centrosomes) (28). In contrast, 74.34% of the PruΔ*ap2X-1* strain exhibited single centrosomes, indicating that most parasites were arrested in the G1 phase and had differentiated into bradyzoites (Fig. 5G).

### AP2X-1 silences sexual stage-specific gene expression

During sexual development, bradyzoites undergo merogony, producing merozoites that further develop into macrogametocytes and microgametocytes (3, 4, 9). After fertilization, felines shed oocysts into the environment, which subsequently differentiate into sporulated oocysts containing eight sporozoites (3, 4, 9). Since AP2X-1 influences the function of the HDAC3/MORC complex, which is known to silence sexual commitment and inhibit the expression of the merozoite- and oocyst-specific genes (11, 19), we assessed the transcriptional effects of *ap2X-1* deletion on the sexual stage regulation.

**Fig 6:**
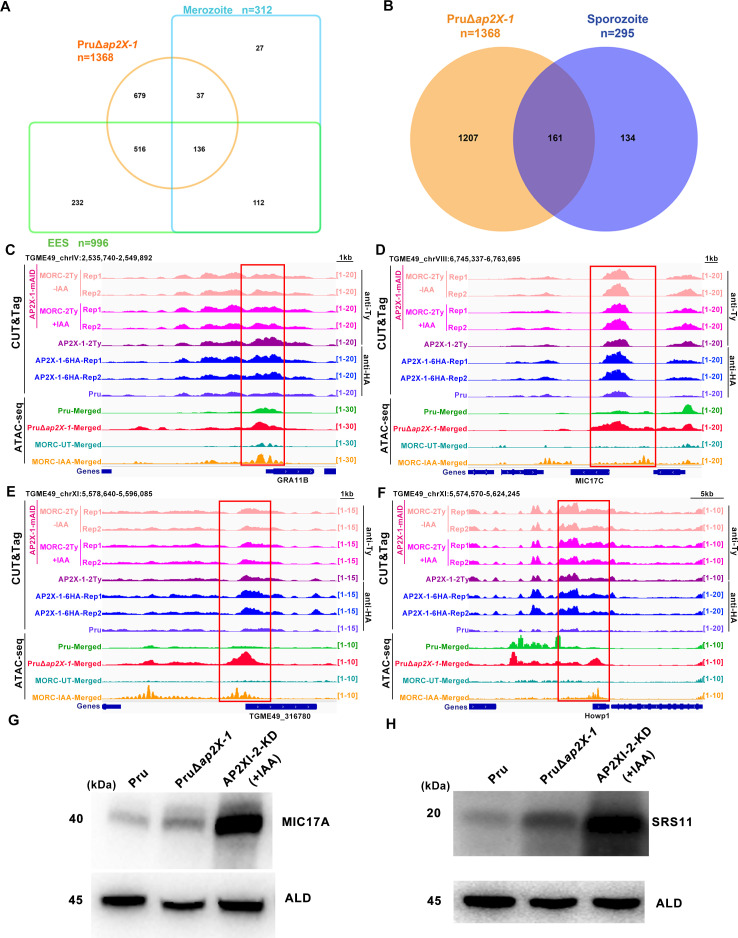
Knockout of *ap2X-1* induces the transcription of sexual stage genes. (**A**) Venn diagram illustrates that knockout of *ap2X-1* leads to the upregulation of genes associated with merozoites and early sexual stages. Data for these genes were obtained from ToxoDB. (**B**) Venn diagram shows that disruption of *ap2X-1* upregulated the sporozoite-associated genes. Data for sporozoite-associated genes was obtained from ToxoDB. (**C and D**) Integrated Genome Viewer (IGV) screenshots of representative merozoite-associated genes (*GRA11B* and *MIC17C*) highlight CUT&Tag signal occupancy for AP2X-1-mAID-MORC-2Ty strain under untreated and post-depletion conditions, as well as AP2X-1-2Ty and AP2X-1-6HA strains under normal culture conditions. The AP2X-1-mAID-MORC-2Ty and AP2X-1-2Ty strains were incubated with mouse anti-Ty antibody, and the AP2X-1-6HA strain was incubated with mouse anti-HA antibody. Chromatin accessibility (ATAC-seq) profiles of the indicated strains are also shown. ATAC-seq data from two biological replicates were combined into a single data set for both the Pru and PruΔ*ap2X-1* strains. (**E and F**) Similar to panels **C and D**. IGV screenshots highlight CUT&Tag signal occupancy for representative sporozoite-associated genes (*TGME49_316780* and *Howp1*) under the same conditions and strains, as well as AP2X-1-2Ty and AP2X-1-6HA strains under normal conditions. Chromatin accessibility (ATAC-seq) profiles are also shown for the indicated strains. ATAC-seq data from two biological replicates were combined into a single data set for both the Pru and PruΔ*ap2X-1* strains. The AP2X-1-mAID-MORC-2Ty and AP2X-1-2Ty strains were incubated with mouse anti-Ty antibody, and the AP2X-1-6HA strain was incubated with mouse anti-HA antibody. (**G and H**) Western blotting indicates activation of merozoite-specific proteins MIC17A (**G**) and SRS11 (**H**) in the PruΔ*ap2X-1* strain. The AP2XI-2 knockout strains, which serve as a model for studying *T. gondii* merogony *in vitro*, served as the positive control. Parasite lysates were incubated with anti-MIC17A and anti-SRS11 antibodies, with anti-aldolase (ALD) used as the loading control.

Strikingly, 55.45% of the merozoite-specific transcripts (173 out of 312) were upregulated following *ap2X-1* knockout (Fig. 6A; Data set S2). These genes included classical merozoite proteins such as *GRA11A*/*B*, MIC17 family, *GRA81,* and several other merozoite-restricted surface proteins, which are related to gamete development and fertilization (Fig. S4A) (29). Additionally, 54.58% of the sporozoite-specific genes (161 out of 295), including *SporoSAG* (30) and *Howp1* (11)—genes expressed during early wall formation and typically restricted to sporozoites—were upregulated in the PruΔ*ap2X-1* strain (Fig. 6B; Fig. S4B). Furthermore, 65.46% (652 out of 996) of the genes strictly expressed during the early entero-epithelial stage (EES1-5), encompassing early merogony to late sexual stages (31), were significantly upregulated in the PruΔ*ap2X-1* strain (Fig. 6A).

CUT&Tag data analysis revealed that the AP2X-1 and MORC were enriched at the promoter regions of some sexual stage-specific genes, and ATAC-seq demonstrated that loss of *ap2X-1* resulted in modestly increased chromatin accessibility, leading to the transcriptional activation of these genes. The ATAC-Seq result was consistent with the effects observed after MORC depletion (25) (Fig. 6C through F). To further validate these findings, western blotting and IFA were performed on the PruΔ*ap2X-1* strain using rabbit-anti-MIC17A and rabbit anti-SRS11 antibodies. The results confirmed that the loss of AP2X-1 indeed upregulates the merozoite proteins MIC17A and SRS11 (Fig. 6G and H), although the SRS11 protein was undetectable by IFA (Fig. S4C and D). These findings suggest that the deletion of *ap2X-1* can upregulate the transcription of sexual stage-specific genes, albeit to a lesser extent than the depletion of MORC or inhibition of HDAC3.

### Knockout of *ap2X-1* silences genes highly expressed in tachyzoites and regulates several secondary transcriptional regulators

**Fig 7:**
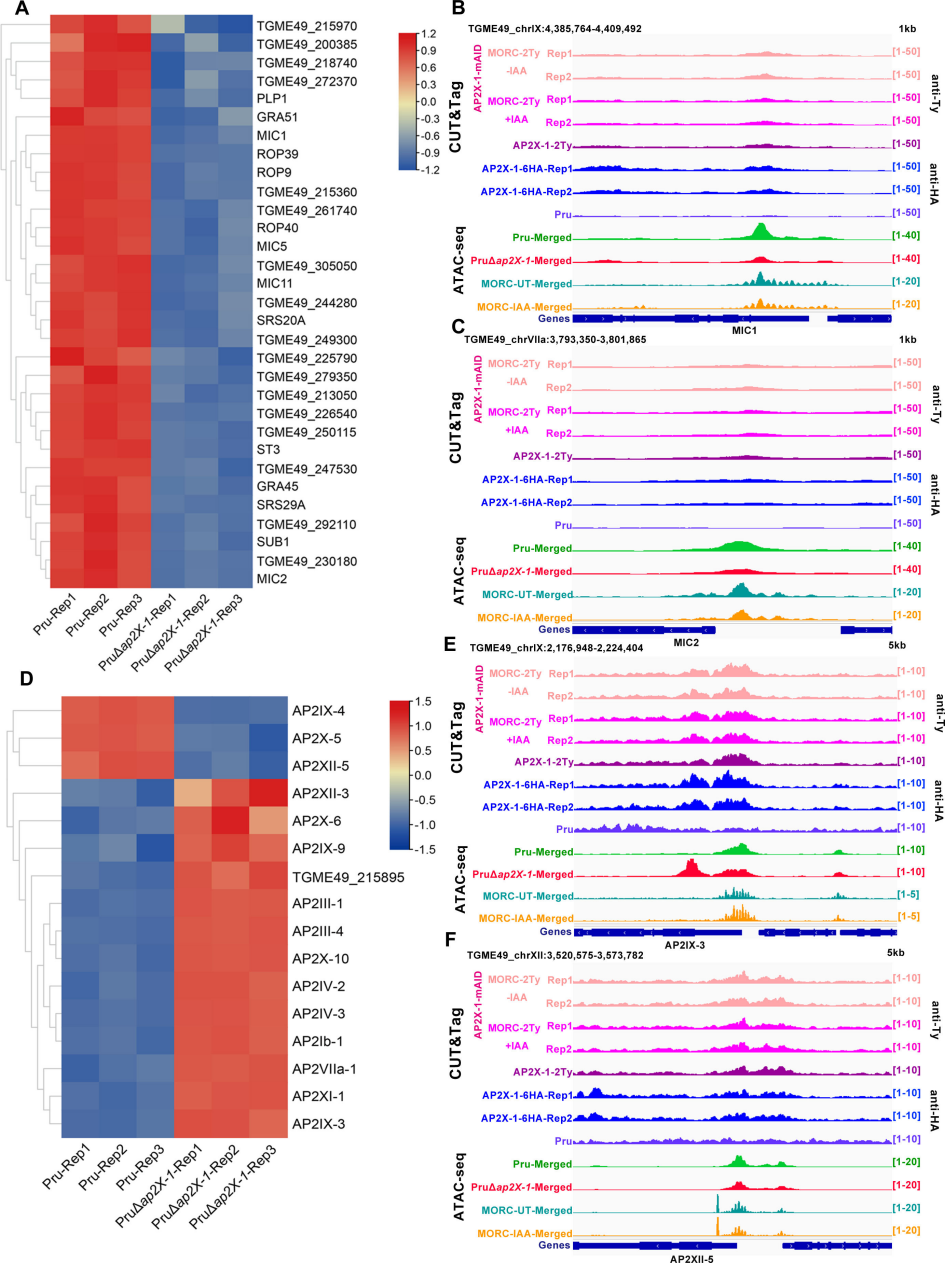
Knockout of *ap2X-1* silences the transcription of tachyzoite genes and regulates several secondary transcriptional regulators under unstressed conditions. (**A**) Heat map illustrates the downregulation of tachyzoite-associated genes following the knockout of *ap2X-1*. The color scale represents log_2_-transformed fold changes. (**B and C**) Integrated Genome Viewer (IGV) screenshots of representative tachyzoite-associated genes (*MIC1* and *MIC2*) highlight CUT&Tag signal occupancy for AP2X-1-mAID-MORC-2Ty strain under untreated and post-depletion conditions, as well as AP2X-1-2Ty and AP2X-1-6HA strains under normal culture conditions. The AP2X-1-mAID-MORC-2Ty and AP2X-1-2Ty strains were incubated with mouse anti-Ty antibody, and the AP2X-1-6HA strain was incubated with mouse anti-HA antibody. Chromatin accessibility (ATAC-seq) profiles of the indicated strains are also shown. ATAC-seq data from two biological replicates were combined into a single data set for both the Pru and PruΔ*ap2X-1* strains. (**D**) Heat map shows the regulation of several secondary transcriptional regulators after knockout of *ap2X-1*. The color scale indicates log_2_-transformed fold changes. (**E and F**) IGV screenshots of representative secondary AP factors (*AP2IX-3* and *AP2X-6*) highlight CUT&Tag signal occupancy for AP2X-1-mAID-MORC-2Ty strain under untreated and post-depletion conditions, as well as AP2X-1-2Ty and AP2X-1-6HA strains under normal culture conditions. The AP2X-1-mAID-MORC-2Ty and AP2X-1-2Ty strains were incubated with mouse anti-Ty antibody, and the AP2X-1-6HA strain was incubated with mouse anti-HA antibody. Chromatin accessibility (ATAC-seq) profiles of the indicated strains are also shown. ATAC-seq data from two biological replicates were combined into a single data set for both the Pru and PruΔ*ap2X-1* strains.

Depletion of MORC or inhibition of HDAC3 has been shown to silence a subset of genes highly expressed in tachyzoites, likely due to secondary transcriptional regulators (11). Similar results were observed in AP2X-1 knockout parasites, where genes, such as the *MIC1*, *MIC2*, *ROP39,* and *GRA45* (29), which are normally highly expressed in tachyzoites, were downregulated in the absence of AP2X-1 (Fig. 7A). Similar to the depletion of AP2XII-1 or AP2XI-2 (20, 22, 23), a reduction in ATAC-seq signal was observed following AP2X-1 knockout and MORC depletion (25), but no clear binding of AP2X-1 to their transcription start sites was detected. This suggests that these tachyzoite-specific genes may be indirectly regulated by AP2X-1 (Fig. 7B and C).

Additionally, *ap2X-1* knockout caused dysregulation of several AP2 factors, with three factors (AP2X-5, AP2XII-5, and AP2IX-4) showing decreased expression, while 13 AP2 factors exhibited increased expression (Fig. 7D). Interestingly, no specific DNA motifs were identified for AP2X-1 binding of these AP2 factors, which are consistent with the lack of a canonical AP2 domain. Among the upregulated AP2 factors, three upregulated AP2 factors (AP2VIIa-1, AP2IV-3, and AP2IV-2) were exclusively expressed in the merozoite stage, while six AP2 factors (AP2X-10, AP2IX-3, AP2III-4, AP2III-1, AP2X-6, and AP2Ib-1) were predicted to guide micro- and/or macrogamete development. Additionally, three AP2 factors (AP2XI-1, AP2XII-3, and TGME49_215895, not yet assigned a formal AP2 number) were highly expressed in the sporozoite stage, and AP2IX-9 was strictly expressed in nuclei of early bradyzoites. Furthermore, the promoters of *AP2III-4*, *AP2XII-5,* and *AP2IX-3* genes exhibited co-binding of AP2X-1 and MORC (Fig. 7E and F). The loss of AP2X-1 or the deletion of MORC increased chromatin accessibility, leading to the transcriptional activation of *AP2IX-3*. To confirm this, we examined the expression of AP2III-4 protein in the AP2X-1 knockout parasites using IFA. Unexpectedly, the AP2III-4 protein expression was not detected in the PruΔ*ap2X-1* strain, but it was detected in the Pru-MORC-mAID-6HA strain treated with IAA (Fig. S4E). Further detection of the AP2III-4 expression in the PruΔ*ap2X-1* strain by real-time quantitative PCR (RT-qPCR) confirmed the increased transcript level of AP2III-4 after AP2X-1 deletion (Fig. S4F). These results suggest that the induction of AP2III-4 expression following AP2X-1 deletion is less pronounced compared to its upregulation upon MORC depletion. It is also possible that AP2X-1 works synergistically with other AP2 factors to influence the function of the HDAC3/MORC complex and repress sexual commitment.

### The virulence of the *ap2X-1* deletion strain is significantly attenuated *in vivo*

**Fig 8:**
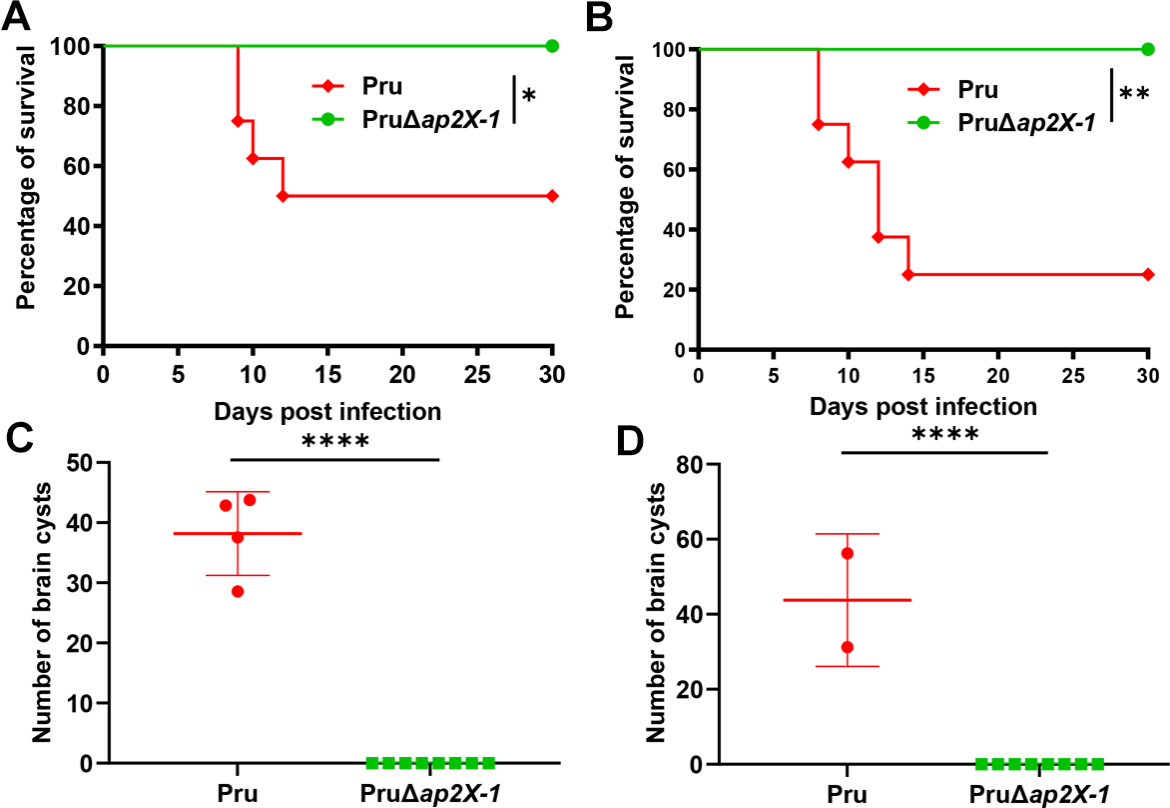
The virulence of PruΔ*ap2X-1* is significantly attenuated *in vivo*. (**A and B**) Survival curves show the survival rates of Kunming mice infected with either 5 × 10^3^ (**A**) or 5 × 10^5^ (**B**) tachyzoites from Pru and PruΔ*ap2X-1* strains. Each group consisted of eight mice, with survival monitored for 30 days. Gehan-Breslow-Wilcoxon test was used to analyze the survival data, *P < 0.05 and **P < 0.01. (**C and D**) The parasite cyst burdens were evaluated based on the number of cysts detected by DBA staining in one cerebral hemisphere of mouse brains that survived for 30 days post-infection in panels **A** and **B**, respectively. Statistical analysis was performed by a two-tailed, unpaired *t*-test, ****P < 0.0001.

Given the crucial role of AP2X-1 in tachyzoite growth *in vitro*, we investigated whether AP2X-1 is essential for parasite infection in mice. Mice were intraperitoneally infected with 5 × 10^3^ and 5 × 10^5^ tachyzoites of wild-type Pru strain and PruΔ*ap2X-1* strain, and their survival was monitored over 30 days. As expected, 50% of the mice infected with 5 × 10^3^ wild-type Pru strain survived (Fig. 8A), while only 25% of those infected with 5 × 10^5^ wild-type Pru strain survived (Fig. 8B). In sharp contrast, all mice infected with PruΔ*ap2X-1* survived without exhibiting any obvious clinical signs (Fig. 8A and B). Brain tissue cysts were evaluated in the surviving mice. All mice infected with the wild-type Pru strain produced brain cysts (Fig. 8C and D), whereas no cysts were detected in mice infected with PruΔ*ap2X-1* (Fig. 8C and D). These results indicate that AP2X-1 is indispensable for parasites’ virulence and the establishment of chronic infection.

## DISCUSSION

As a highly successful parasite, *T. gondii* has developed a remarkable ability to reprogram its gene expression, enabling it to adapt to the hostile microenvironment encountered within host cells. However, the precise underlying mechanisms remain poorly understood (11). The AP2 factors play a pivotal role in regulating genes involved in *T. gondii*’s growth and stage transformation (11–18, 20–23). In this study, we characterized a member of the AP2 family, AP2X-1, which was localized to the nucleus in both tachyzoite and bradyzoite stages. Deletion of the *ap2X-1* gene significantly affected tachyzoite growth, leading to the increased expression of genes associated with bradyzoite and sexual development.

MORC, a key regulatory protein, has been reported to associate with at least 12 AP2 factors, recruiting HDAC3 to restrict chromatin accessibility to genes that are exclusively expressed during the bradyzoite and sexual stages of *T. gondii* (11, 19–21). This complex plays a crucial role in regulating *T. gondii*’s ability to switch between developmental stages. Interestingly, AP2X-1 associates with the HDAC3/MORC complex (11, 19–21). Co-immunoprecipitation (Co-IP) assays confirmed that AP2X-1 associates with the HDAC3/MORC complex. The loss of the *ap2X-1* induced bradyzoite differentiation. Transcriptome analysis revealed that many bradyzoite-specific genes were significantly upregulated in the PruΔ*ap2X-1* strain, including the bradyzoite markers such as *BAG1*, *LDH2*, and the cyst wall protein *CST1*. Additionally, numerous pre-sexual and sexual stage-specific genes, such as *GRA11A/B*, *GRA81*, and *Howp1*, were also upregulated in the PruΔ*ap2X-1* strain.

CUT&Tag and ATAC-seq analyses demonstrated that AP2X-1-associated regions are located near the transcriptional start sites of most bradyzoite- and sexual stage-specific genes, such as *LDH2*, *SRS9*, *GRA11B*, *MIC17C,* and *Howp1*, with significant overlap (81.30%) in regions targeted by the HDAC3/MORC complex. This suggests that AP2X-1 may influence the function of the HDAC3/MORC complex to alter chromatin compaction and accessibility, thereby repressing the expression of genes associated with bradyzoite and sexual stages in *T. gondii*. Interestingly, while certain sexual stage-specific genes were upregulated in the *ap2X-1* knockout strain, some of their expression was not detected by IFA. This indicates that the level of gene induction from *ap2X-1* disruption is less pronounced compared to MORC depletion or HDAC3 inhibition. Thus, while AP2X-1 shares a regulatory pattern with the HDAC3/MORC complex, its repression effect is somewhat less potent.

Several AP2 factors are known to form homodimers or heterodimers with other proteins, influencing DNA-binding specificity and affinity. For instance, AP2XI-2 and AP2XII-1 cooperatively bind to DNA as a heterodimer to selectively and synergistically suppress the expression of merozoite-specific genes. Co-depletion of these factors induces parasites to undergo merogony and produce mature merozoites (20, 22, 23). Based on this, we hypothesized that AP2X-1 may work synergistically with other factors, such as AP2XII-2 or AP2XII-5 (identified as a partner of MORC) (21, 32), to repress bradyzoite- and sexual stage-specific gene expression. This may explain why AP2X-1 lacks a definitive AP2 domain, although the presence of other novel, uncharacterized DNA-binding domains cannot be excluded.

In alignment with the roles of the HDAC3/MORC complex, several tachyzoite-specific genes were also downregulated in the absence of AP2X-1. This suggests that secondary transcriptional regulators are also activated to guide these changes. Indeed, knockout of *ap2X-1* dysregulated the expression of 16 secondary AP2 factors, with three AP2 factors downregulated, likely acting as transcriptional activators in tachyzoites (11). On the other hand, 13 AP2 factors were upregulated in the absence of AP2X-1, with eight directly targeted by AP2X-1 and MORC. This supports the hypothesis that MORC depletion initiates a downstream network of secondary transcriptional regulators, guiding developmental trajectories. For example, AP2IX-9, a bradyzoite transcriptional repressor, is upregulated in the *ap2X-1* deletion strain, potentially preventing the differentiation of merozoite back into bradyzoite (16). AP2X-10, the most abundant AP2 factor in oocysts, is typically expressed in the enteroepithelial and oocyst stages, possibly guiding micro- and/or macrogamete development (11, 21). The findings from our present study suggest that AP2X-1 regulates the repression of AP2X-10, positioning AP2X-1 as a higher-level regulator in the transcriptional regulatory cascade that governs *T. gondii* sexual development. The knockout of *ap2X-1* or the depletion of MORC triggers the hierarchical expression of secondary AP2 factors, many of which are primarily active during the bradyzoite and sexual stages. These secondary factors likely function as downstream activators or repressors that help guide sexual commitment. Furthermore, this division of roles between primary and secondary AP2 factors enables a highly coordinated and specific transcriptional response, ensuring the unidirectional progression of *T. gondii* life cycle.

In conclusion, this study provides new insight into the role of AP2X-1 in sexual development in *T. gondii.* Our findings show that knockout or depletion of AP2X-1 significantly impairs *T. gondii* growth and alters developmental progression. We further suggest that AP2X-1 plays an important role in influencing the function of the HDAC3/MORC complex, which represses the expression of genes linked to bradyzoite and sexual stages. However, a direct mechanism by which AP2X-1 recruits or regulates the HDAC3/MORC complex has not been demonstrated. Although our data indicate that loss of AP2X-1 modestly reduces MORC association at target promoters and leads to increased chromatin accessibility, alternative explanations cannot be excluded. For example, loss of AP2X-1 might indirectly affect HDAC3/MORC complex stability, transcription, or its recruitment via additional factors. Thus, while AP2X-1 appears to modulate the HDAC3/MORC complex’s repressive functions, the precise mechanisms underlying this modulation remain to be clarified. Further studies are needed to determine whether AP2X-1 acts directly or indirectly to influence HDAC3/MORC complex targeting and gene silencing. Overall, these results establish AP2X-1 as a key factor that negatively regulates sexual commitment in *T. gondii* through its association with the HDAC3/MORC complex. The identification of AP2X-1 provides an important foundation for future investigations into the regulatory mechanisms governing sexual development in *T. gondii*.

## MATERIALS AND METHODS

### Cell culture and parasite strains

Human foreskin fibroblast cells, originally purchased from the American Type Culture Collection (ATCC SCRC-104), were maintained in Dulbecco’s modified Eagle medium (DMEM; Gibco) supplemented with 10% fetal bovine serum (FBS; Gibco), 100 mg/mL streptomycin, and 100 U/mL penicillin, under 37°C with 5% CO_2_ as described previously (33). The tachyzoites of *T. gondii*, including PruΔ*ku80*Δ*hxgprt* (referred to as Pru), PruΔ*ku80*Δ*hxgprt*::TIR1-3Flag (referred to as Pru-TIR1), and the knockout of *ap2X-1* strains, were propagated in confluent monolayers of HFFs grown in DMEM supplemented with 2% FBS (22). When the HFF cells contain many PVs fully packed with tachyzoites, the tachyzoites are released mechanically from the HFF cells by passing through a 27-gauge needle. The freshly isolated tachyzoites were filtered using a 5 µm polycarbonate membrane filter to remove host cell debris (22).

### Mice

Six- to eight-week-old female Kunming mice were obtained from the Center of Laboratory Animals of Lanzhou Veterinary Research Institute. Mice were acclimated to the environment for at least 1 week before the start of the experiment (22). All animal experiments were performed according to the approved institutional and local guidelines.

### Construction of *ap2X-1* knockout strain

CRISPR-Cas9-mediated gene homologous recombination was used to delete AP2X-1 as previously described (34). Briefly, specific CRISPR plasmids of the corresponding AP2X-1 were constructed by replacing the UPRT targeting gRNA in pSAG1::Cas9-U6::sgUPRT with SgRNA of AP2X-1. To construct 5′UTR-DHFR-3′UTR plasmids of the corresponding AP2X-1, the 5′ and 3′ homologous arms of genes flanking *ap2X-1* were amplified from *T. gondii* genomic DNA, and the DHFR fragment was amplified from pUPRT-DHFR-D plasmid. The three fragments were then ligated into the pUC19 plasmid using a Clone Express II one-step cloning kit (Vazyme, Nanjing, China) as previously described (22). The corresponding gene-specific CRISPR plasmid (40 µg) and the homologous template fragment of 5′UTR-DHFR-3′UTR (25 µg) were co-transfected into freshly egressed tachyzoites. After selection with 3 µM pyrimethamine and cloning by limiting dilution, the single PruΔ*ap2X-1* mutant was identified by PCR. All the primers used in the study are listed in Table S1.

### Generation of the C-terminal-tagged strain

For C-terminal tagging, we constructed CRISPR-Cas9 plasmids that create a double-strand nick in the 3-untranslated region (3-UTR) of the gene of interest close to the stop codon as previously described (22, 35). The plasmids were co-transfected with amplicons flanked with short homology regions containing a 6HA (6× hemagglutinin)-DHFR, mAID-HA-HXGPRT, or 2Ty-HXGPRT into the corresponding strain. The successful insertion of 6HA, mAID-HA, or 2Ty tag was verified by PCR and sequencing. For knockdown, parasites were treated with 500 µM 3-indoleacetic acid (1:1,000 dilution), while the control parasites were mock treated with 0.1% ethanol only (35). The strain with auxin-induced depletion of MORC and the strain with FR235222-induced inhibition of HDAC3 were described in previous research (11).

### Complementation of the knockout strains

To complement the PruΔ*ap2X-1* knockout strain, a wild-type copy of AP2X-1 with a C-terminal 2Ty epitope tag, driven by its native promoter, was inserted into the *hxgprt* locus of the PruΔ*ap2X-1* strain using CRISPR-Cas9 (36). To construct the complemented plasmid, the *ap2X-1* promoter fragment was amplified from *T. gondii* Pru DNA, and the coding sequence of *ap2X-1* was amplified from cDNA. These components were then inserted into the pSAG1-CAT plasmid to generate the pAP2X-1-AP2X-1-2Ty-CAT plasmid. The validated plasmid was used as a template to amplify the insertional cassette with primers containing homology segments to the *hxgprt* locus of *T. gondii.* The pSAG1:CAS9-U6-Sg*hxgprt* vector was co-transfected with the insertional cassette into PruΔ*ap2X-1* tachyzoites. After several cycles of chloramphenicol selection, independent clones PruΔ*ap2X-1*C obtained by modified limiting dilution were confirmed by PCR, IFA, and western blotting.

### Immunofluorescence assay

The parasite-infected HFF cells were fixed with 4% paraformaldehyde (PFA) in phosphate-buffered saline (PBS) for 20 min, permeabilized with 0.1% Triton X-100 in PBS for 15 min, and then blocked with 3% bovine serum albumin in PBS for 2 h. The samples were incubated with primary antibodies, including rabbit anti-IMC1 (1:500 dilution), rabbit anti-BAG1 (1:500 dilution), rabbit anti-MIC17A (1:500 dilution), rabbit anti-SRS11 (1:500 dilution), rabbit anti-Centrin-1 (1:500 dilution, available in our laboratory), mouse anti-HA (1:500 dilution), or mouse anti-Ty (1:1,000 dilution, Invitrogen, USA), at 37°C for 2 h or at 4°C overnight, followed by washing three times with PBS. Subsequently, the samples were incubated with secondary antibodies, including goat anti-rabbit IgG (H + L) antibody conjugated with Alexa Fluor 488 (1:500 dilution) and goat anti-mouse IgG (H + L) antibody conjugated with Alexa Fluor 594 (1:500 dilution, Invitrogen, USA) for 1 h at 37°C. For the localization of AP2X-1 in bradyzoites, the secondary antibodies used were donkey anti-rabbit IgG (H + L) antibody conjugated with Alexa Fluor 647 (1: 500 dilution), goat anti-mouse IgG (H + L) antibody conjugated with Alexa Fluor 594 (1:500 dilution, Invitrogen, USA), and *Dolichos biflorus agglutinin* (1:500 dilution, Vector Laboratories, USA). Nucleus was stained with 4′,6-diamidino-2-phenylindole (1:1,000 dilution, available in our laboratory), and images were captured using a Leica confocal microscope (TCS SP8, Leica, Germany), as previously described (22).

### Western blotting

Parasites growing in confluent HFF cells were mechanically released and filtered to remove host cell debris, followed by centrifugation at 2,000 × *g* for 10 min. The purified tachyzoite pellets were lysed with radioimmunoprecipitation assay lysis buffer containing protease and phosphatase inhibitor cocktail on ice for 1 h. The lysate was centrifuged at 1,000 × *g* for 10 min, and the protein supernatant was collected for subsequent analysis. The supernatant was mixed with 4× sample loading buffer and boiled at 100°C for 20 min. The protein samples were separated by sodium dodecyl sulfate-polyacrylamide gel electrophoresis (SDS-PAGE) on 12% polyacrylamide gels and were transferred to polyvinylidene fluoride (PVDF) membranes using semi-dry rotating electroblotting. The membranes were blocked in 5% non-fat milk and incubated in primary antibodies, rabbit anti-HA diluted 1:1,000 (Invitrogen, USA), rabbit anti-MIC17A (1:1,000 dilution), rabbit anti-SRS11 (1:1,000 dilution), rabbit anti-BAG1 (1:1,000 dilution), rabbit anti-Aldolase (1:500 dilution, available in our laboratory), mouse anti-Ty (1:5,000 dilution, Invitrogen, USA) at 37°C for 1 h. This was followed by incubation in secondary antibodies: goat anti-rabbit IgG (H + L) HRP diluted 1:5,000 and goat anti-mouse IgG (H + L) HRP diluted 1:5,000 (Biodragon, China) at 37°C for 1 h. The PVDF membrane was incubated with Pierce enhanced chemiluminescence (Thermo Fisher Scientific, USA) to visualize the proteins on a ChemiDoc XRS+ (Bio-Rad, USA), as previously described (36).

### Effect of AP2X-1 deletion on *Toxoplasma* growth

The effect of *ap2X-1* deletion on the growth of tachyzoites was evaluated using a standard plaque assay. Briefly, approximately 200 freshly harvested tachyzoites of the Pru, PruΔ*ap2X-1,* and PruΔ*ap2X-1*C strains were inoculated into confluent HFF monolayers grown in 12-well tissue culture plastic plates (Thermo Fisher Scientific, USA). The plates were then incubated at 37°C in a humidified environment with 5% CO_2_. For conditional depletion of the TIR1 and AP2X-1-mAID strains, either 500 µM IAA or 0.1% ethanol was added to the medium before introducing the tachyzoites. After 9 days of incubation, the culture medium was discarded, and the infected cells were fixed with 4% PFA for 30 min. To visualize plaques (areas devoid of host cells), fixed cells were stained with 0.5% crystal violet in PBS for 30 min at ambient temperature. The size and number of each plaque were determined as previously described (22). The experiments were carried out three times, each with three technical replicates.

### Invasion, intracellular replication, and egress assays

To assess the *T. gondii* invasion efficiency in the absence of AP2X-1, ~5 × 10^6^ freshly harvested tachyzoites of the Pru, PruΔ*ap2X-1,* and PruΔ*ap2X-1*C strains were inoculated into HFFs cultured in 12-well tissue culture plates for 1 h, and the cells were then fixed with 4% PFA. For conditional depletion of AP2X-1-mAID strains, parasites were grown for 48 h with or without IAA, then the freshly purified tachyzoites suspended in DMEM medium with or without IAA were added to HFFs cultured in 12-well tissue culture plates for 1 h, and the cells were then fixed with 4% PFA. The samples were then incubated with mouse anti-SAG1 (1:500 dilution) at 37°C for 1 h and washed three times with PBS. Subsequently, the samples were incubated with goat anti-mouse IgG (H + L) antibody conjugated with Alexa Fluor 594 (1:500 dilution) for 1 h at 37°C, followed by three washes with PBS. After permeabilization with 0.1% Triton X-100 in PBS, a second round of immunolabeling was performed using a primary antibody, rabbit anti-IMC1 (1:500 dilution) for 1 h at 37°C, and a secondary antibody, goat anti-rabbit IgG (H + L) antibody conjugated with Alexa Fluor 488 (1:500 dilution) for 1 h at 37°C. The tachyzoites that failed to invade the host cells and remained extracellular were stained red, while the intracellular and extracellular tachyzoites were stained green (37, 38). The parasite invasion efficiency was determined by calculating the ratio of intracellular parasites to the total number of tachyzoites observed in 20 microscopic fields per sample for each strain (39). The experiments were carried out three times, each with three technical replicates.

We examined the effect of *ap2X-1* deletion on *T. gondii* replication. Briefly, ~5 × 10^5^ freshly harvested tachyzoites of Pru, PruΔ*ap2X-1,* and PruΔ*ap2X-1*C strains were used to infect HFF cells for 2 h. The uninvaded (extracellular) parasites were washed with warm DMEM, and the parasites were cultured for an additional 34 h. For conditional depletion of the TIR1 and AP2X-1-mAID strains, freshly harvested tachyzoites were used to infect HFFs in 12-well tissue culture plastic plates for 2 h, then the infected cultures were washed and incubated with fresh culture medium, with or without IAA for an additional 34 h. The cells were then fixed with 4% PFA, followed by permeabilization with 0.1% Triton X-100. The samples were then incubated with rabbit anti-IMC1 (1:500 dilution) at 37°C for 2 h and washed three times with PBS. Subsequently, the samples were incubated with goat anti-rabbit IgG (H + L) antibody conjugated with Alexa Fluor 488 (1:500 dilution) for 1 h at 37°C. At least 100 PVs were examined in each well to determine the number of intracellular tachyzoites per PV as previously described (36). The experiments were carried out three times, each with three technical replicates.

The effect of *ap2X-1* deletion on *T. gondii* egress was also examined. Briefly, ~5 × 10^4^ freshly harvested tachyzoites of the Pru, PruΔ*ap2X-1,* and PruΔ*ap2X-1C* strains were inoculated into HFFs cultured in 12-well tissue culture plates for 48–60 h to obtain a similar number of parasites per PV, and the cells were then treated with 3 µM calcium ionophore A23187 in DMEM (preheated at 37°C). After 2 min of egress, the sample was immediately fixed with 4% PFA, followed by permeabilization with 0.1% Triton X-100. For conditional depletion of the TIR1 and AP2X-1-mAID strains, the tachyzoites were used to infect cell monolayers in 12-well tissue culture plastic plates for 48–60 h with or without IAA. The intact PVs were stained with rabbit anti-GRA5, and the parasites were stained with mouse anti-IMC1. Subsequently, the samples were incubated with goat anti-rabbit IgG (H + L) antibody conjugated with Alexa Fluor 488 (1:500 dilution) and goat anti-mouse IgG (H + L) antibody conjugated with Alexa Fluor 594 (1:500 dilution, Invitrogen, USA) for 1 h at 37°C. At least 100 PVs were counted in each well to determine the number of tachyzoites as previously described (22, 40). The experiments were conducted three times, each with three technical replicates.

### Bradyzoite differentiation assay

Bradyzoite differentiation experiments were conducted as previously described (41, 42). Briefly, Pru, PruΔ*ap2X-1,* and PruΔ*ap2X-1*C strains were used to infect a confluent monolayer of HFF cells cultured under normal medium (at 37°C and 5% CO_2_) for 2 days. For the mAID-based strains, the tachyzoites were allowed to infect HFF cells in the presence of 500 µM IAA and the addition of 0.1% ethanol at 37°C, 5% CO_2_ for 2 days. The samples were fixed with 4% PFA and then permeabilized with 0.1% Triton X-100. The samples were subsequently incubated with rabbit anti-IMC1 (1:500 dilution) at 37°C for 2 h and washed three times with PBS. Then, the samples were incubated with DBA and goat anti-rabbit IgG (H + L) antibody conjugated with Alexa Fluor 594 (1:500 dilution) for 1 h at 37°C. The proportion of DBA-positive PVs in 100 randomly selected PVs was determined. The experiments were conducted three times, each with three technical replicates.

### Co-immunoprecipitation and mass spectrometry

Co-IP and mass spectrometry were used to identify AP2X-1 interacting proteins in AP2X-1-2Ty, MORC-2Ty, and HDAC3-2Ty tachyzoites, with Pru tachyzoites being used as a negative control. For each strain, tachyzoites were cultured in normal medium for 3 days, and then the heavily infected cells were washed with chilled PBS and scraped. The intracellular parasites were released from the feeder host cells by syringe lysis and purified by filtration through 5 µm polycarbonate membranes. The filtered parasites were centrifuged at 1,000 × *g* for 10 min, and the resulting pellets were used to extract the total protein. The pellets were lysed using the IP lysis buffer (25 mM Tris HCl, pH 7.4, 150 mM NaCl, 1% NP-40, 1 mM EDTA, and 5% glycerol) for 1 h on ice and then centrifuged at 13,000 rpm for 10 min. The supernatants of the parasite lysates were incubated with 2 µg mouse anti-Ty antibody (Invitrogen, USA) and incubated overnight at 4°C with rotation. The antigen sample/antibody mixture was added to protein A + G magnetic beads (Thermo Fisher Scientific, USA) and incubated for 4 h at 4°C with rotation. Subsequently, the protein-bound magnetic beads were washed four times with IP lysis buffer, and the bound proteins were eluted from the magnetic beads according to the manufacturer’s instructions with low-pH elution (Pierce Class Magnetic IP/Co-IP Kit, Thermo Fisher Scientific, USA). The bound proteins separated by SDS-PAGE were stained with Coomassie blue and identified by liquid chromatography-mass spectrometry as previously described (43). The RAW data were searched using the MaxQuant (version 1.6.1.0) tool against the *T. gondii* ME49 genome in ToxoDB (https://toxodb.org) (44). To ensure the reliability of the results, the peptide and protein identifications were filtered at a false discovery rate (FDR) of 1% at the spectral level (PSM level FDR ≤ 0.01).

### RNA sequencing

Tachyzoites of Pru and PruΔ*ap2X-1* strains cultured in HFF cells of normal medium for 3 days were collected and syringe filtered as described in the Co-IP method above. Total RNA of the parasites was extracted using an RNeasy kit (Qiagen, MD, USA) following the manufacturer’s protocol. The RNA samples were subjected to RNase-free DNase treatment to remove any remaining genomic DNA after extraction. The mRNA was purified by Oligo (dT)-attached magnetic beads and amplified using random hexamer-primed reverse transcription to construct the libraries, which were sequenced on MGISEQ 2000 (BGI-Shenzhen, China). The raw sequencing reads were trimmed, and the clean reads were mapped to the ME49 genome (downloaded from ToxoDB) using the Bowtie2 software. The level of gene expression was determined by the values of the reads per kilobase per million mapped reads (RPKM) method, and the differentially expressed genes (DEGs) were identified by comparing their RPKM values using DESeq2 software (45). Volcano plots were generated using R, utilizing the gene expression levels from various samples. Genes that met the criteria of a log_2_ fold change of ≥1 or ≤−1 and *Q* value of <0.05 were deemed statistically significant. RNA-Seq data pertaining to MORC and HDAC3 (11) impairment were obtained from the GEO under accession number GSE136123.

### Single-cell RNA library preparation and sequencing

Tachyzoites of Pru and PruΔ*ap2X-1* strains were cultured in HFF cells in normal medium for 3 days. Subsequently, approximately 5 × 10^5^ intracellular tachyzoites were harvested and washed twice in PBS supplemented with 0.04% bovine serum albumin (Thermo Fisher). Cells with greater than 80% viability are qualified for library construction. scRNA-seq libraries were generated using the DNBelab C Series Single-Cell Library Prep Set (MGI, 1000021082) (46). Briefly, the single-cell/nucleus suspensions were converted to barcoded scRNA-seq libraries through droplet encapsulation, emulsion breakage, mRNA-captured bead collection, reverse transcription, cDNA amplification, and purification. Indexed sequencing libraries were generated following the manufacturer’s protocols. Library concentrations were determined using the Qubit ssDNA Assay Kit (Thermo Fisher Scientific, Q10212). Libraries were sequenced using the DIPSEQ T7 sequencer (BGI Shenzhen, China) (47).

### Analysis of single-cell RNA sequencing data

The raw gene expression matrix for each sample was generated using DNBelab_C4scRNA (version 1.0.1) (48). Downstream analysis was performed using the Seurat package (version 3.2.0) in R (49). Quality control was employed according to the number of detected genes and the proportion of mitochondrial reads per cell. In particular, cells with fewer than 200 detected genes or >90% of the maximum genes were filtered out. For the mitochondrial metric, the top 15% of cells with the highest mitochondrial read ratio were filtered out. Potential doublets were identified and removed by DoubletDetection (50). Cell cycle analysis was conducted with the Cell Cycle Scoring function in the Seurat program. The gene expression data set was normalized, and principal component analysis was performed using only the 2,000 highly variable genes in the data set. Raw sequencing reads were trimmed, and the clean reads were mapped to the ME49 genome (downloaded from ToxoDB). U-MAP was then used for two-dimensional visualization of the resulting clusters. Marker genes for each cluster were identified using the FindAllMarkers function in the Seurat package with the following parameters: log fold change (logFC) threshold > 0.25, minimum percentage of cells expressing the gene (min.pct) > 0.1, and adjusted *P*-value (*P*adj) ≤ 0.05. Subsequently, cell clusters were annotated to known cell types using the SCSA method (51). DEGs between samples were identified using the FindMarkers function in Seurat with the same parameter settings: logFC > 0.25, min.pct > 0.1, and *P*adj ≤ 0.05. The specific genes in the four stages of *T. gondii* selected as marker genes include bradyzoite stage genes (*TGME49_291040* [*LDH2*], *TGME49_320190* [*SRS9*], *TGME49_264660* [*CST1*], *TGME49_202020* [*DnAK-TPR*], *TGME49_268860* [*Enolase 1*], *TGME49_259020* [*BAG1*], *TGME49_208730* [*MCP4*], *TGME49_209755* [*MAG2*], and *TGME49_306620* [*AP2IX-9*]), merozoite stage genes (*TGME49_200230* [*MIC17C*], *TGME49_200240* [*MIC17B*], *TGME49_237800* [*GRA11B*], *TGME49_212410* [*GRA11A*], *TGME49_277230* [*GRA82*], *TGME49_273980* [*GRA80*], *TGME49_207010* [*SRS48K*], *TGME49_243940* [*GRA81*], and *TGME49_207005* [*SRS48Q*]), sporozoites stage genes (*TGME49_210950* [*OWP*], *TGME49_258550* [*SRS28*], and *TGME49_316890* [*Howp1*]), and tachyzoite stage genes (*TGME49_201780* [*MIC2*], *TGME49_291890* [*MIC1*], *TGME49_270250* [*GRA1*], *TGME49_227620* [*GRA2*], and *TGME49_309590* [*ROP1*]).

### Cleavage under targets and tagmentation assay

To investigate chromatin dynamics, the CUT&Tag assay was performed using the Hyperactive Universal CUT&Tag Assay Kit for Illumina (Vazyme Biotech, #TD903, China) as previously described (52, 53). Briefly, ~1 × 10^7^ intracellular tachyzoites were harvested, washed twice with wash buffer, and nuclei were extracted with the pre-cooled NE Buffer before they were bound to ConA beads (concanavalin A-coated magnetic beads) for 10 min at room temperature. After that, the parasite nuclei of the AP2X-1-6HA strain were incubated with mouse anti-HA (1:50 dilution) at 4°C overnight, and parasite nuclei of AP2X-1-mAID-MORC-2Ty with or without IAA and AP2X-1-2Ty strains were incubated with mouse anti-Ty at 4°C overnight. The next day, anti-mouse IgG (1:100 dilution) was added and incubated for 1 h at room temperature. Then, parasite nuclei were washed three times with Dig-wash buffer and incubated with 0.04 µM pA/G-Tnp for 1 h at room temperature. Similarly, parasite nuclei were washed three times with Dig-300 buffer, resuspended in tagmentation buffer, and incubated at 37°C for 1 h in PCR. Tagmentation was stopped by adding proteinase K, buffer L/B, and DNA extraction beads. After incubation at 55°C for 10 min in PCR, samples were plated on a magnet, and the unbound liquid was removed. Beads were gently rinsed twice with 80% ethanol, and DNA was eluted with double-distilled water. The parasite nuclei were processed to construct libraries using TruePrep Index Kit version 2 for Illumina from Vazyme Biotech (#TD202, China). The AP2X-1-mAID-MORC-2Ty strain (with or without IAA treatment) and AP2X-1-6HA strain were analyzed for two biological replicates. Libraries were sequenced on the Illumina Novaseq platform at Novogene Science and Technology Co., Ltd (Beijing, China). Clean reads were aligned to the ME49 genome using BWA (version 0.7.12) (54). The peak calling was performed with MACS2 (version 2.1.0) (55), and the nearest genes around the peak, as well as annotations of the genomic region of the peak, were analyzed using ChIPseeker (56). The peak distribution along the genomic regions of the genes of interest was visualized using Integrative Genomics Viewer (IGV) (57).

### Assay for transposase-accessible chromatin with high-throughput sequencing

The ATAC-seq assay was performed using the Hyperactive ATAC-Seq Library Prep Kit for Illumina (Vazyme Biotech, #TD701, China) as previously described (24). Briefly, approximately 1 × 10^8^ intracellular tachyzoites (Pru and PruΔ*ap2X-1*) were harvested from HFF cell monolayers grown in a T75 tissue culture flask, which was freshly scraped, gently passed through a 27-gauge needle, and centrifuged at 500 × *g*. The pellet was washed twice with pre-cooled TW Buffer and lysed with pre-cooled lysis buffer on ice for 1 h to prepare the nuclei. After that, the nuclei were then resuspended in pre-cooled tagmentation Mix (TW Buffer, 10% Tween 20, 1% Digitonin, Nuclease-free ddH_2_O, 5× TTBL, and TTE Mix V50) and incubated at 37°C for 30 min in a water bath. Tagmentation was stopped by adding Stop Buffer and incubating the sample at room temperature for 5 min. The nuclei were then incubated with ATAC DNA Extract Beads at room temperature for 5 min, after which the samples were plated on a magnet rack to remove the unbound liquid. Beads were gently rinsed twice with 80% ethanol, and DNA was eluted with double-distilled water. Then, the parasite nuclei were processed to construct libraries using TruePrep Index Kit version 2 for Illumina from Vazyme Biotech (#TD202, China). The sample clustering was conducted on a cBot Cluster Generation System using TruSeq PE Cluster Kit v3-cBot-HS (Illumina) following the manufacturer’s recommendations (PE150). Libraries were sequenced on the Illumina Novaseq Platform at Novogene Science and Technology Co., Ltd (Beijing, China). Clean reads were aligned to the ME49 genome using BWA (version 0.7.12) (54). The peak calling was conducted using MACS2 (version 2.1.0) (55), and the nearest genes around the peak, as well as annotation of the genomic region of the peak, were analyzed using ChIPseeker (56). The peak distribution along the genomic regions of the genes of interest was visualized using IGV (57). ATAC-seq data for MORC-depleted parasites were obtained from the GEO database under the accession number GSE271901 (25).

### Real-time quantitative PCR

To compare transcript levels between wild-type Pru and PruΔ*ap2X-1* strains, total RNA was extracted from parasites using the RNeasy Kit (Qiagen, MD, USA) according to the manufacturer’s instructions. RNA samples were then processed using the PrimeScript RT Master Mix and TB Green Premix Ex Taq II (Takara, Japan). RT-qPCR was performed using the QuantStudio 5 Real-Time PCR System. Expression levels of target genes were normalized to *β-tubulin* and subsequently compared. Each sample was analyzed independently in triplicate. All primers are listed in Table S1.

### Virulence and cyst burden in infected mice

To assess the virulence of PruΔ*ap2X-1*, Kunming mice (eight mice/group) were injected intraperitoneally with 5 × 10^3^ or 5 × 10^5^ tachyzoites of Pru and PruΔ*ap2X-1* strains. Mice were monitored twice daily for up to 30 days to observe any symptoms of infection and determine humane endpoints. To assess the cyst burden, the surviving mice were sacrificed by CO_2_ asphyxiation at 30 days post-infection, and the number of cysts in brain tissues was counted, as previously described (34).

### Statistical analyses

All statistical analyses were conducted using GraphPad Prism (GraphPad Software, La Jolla, CA, USA). Significant differences between two groups were assessed using a two-tailed, unpaired *t*-test, while comparisons among three or more groups were performed using one-way analysis of variance. *P* values < 0.05 were considered statistically significant.

## Data Availability

The CUT&Tag and ATAC-Seq data generated in this study have been deposited to the Gene Expression Omnibus (GEO) database under the accession number GSE249123. The RNA-Seq and scRNA-seq data generated in this study have been deposited to the short read archive (SRA) of NCBI under the accession number PRJNA1047766. The mass spectrometry proteomics data of AP2X-1 interactome have been deposited in the ProteomeXchange Consortium via the PRIDE partner repository with the data set identifier PXD065585.

## SUPPLEMENTAL MATERIAL

Data set S1 mbio.00052-25-s0001.xlsx Summary of the mass spectrometry analysis of *Toxoplasma gondii* proteins co-immunoprecipitated with Pru, AP2X-1-2Ty, MORC-2Ty and HDAC3-2Ty strains.

Data set S2 mbio.00052-25-s0002.xlsx Summary of the transcriptomic analysis of the Pru and PruΔ*ap2X-1* strains under normal culture condition.

Data set S3 mbio.00052-25-s0003.xlsx Summary of the transcriptomic data analysis of the AP2X-1-mAID-HA strains under normal culture conditions with or without IAA.

Data set S4 mbio.00052-25-s0004.xlsx Summary of peak calls from CUT&Tag for AP2X-1-2Ty, AP2X-1-6HA, and AP2X-1-mAID-MORC-2Ty strains with or without IAA.

Data set S5 mbio.00052-25-s0005.xlsx Summary of peak calls from ATAC-seq for Pru and PruΔ*ap2X-1* strains.

Supplemental figures mbio.00052-25-s0006.pdf Figures S1 to S4.

Table S1 mbio.00052-25-s0007.docx Primers used in the study.

ASM does not own the copyrights to Supplemental Material that may be linked to, or accessed through, an article. The authors have granted ASM a non-exclusive, world-wide license to publish the Supplemental Material files. Please contact the corresponding author directly for reuse.
