# Supplementary material for: AP2X-1 is a negative regulator of Toxoplasma gondii sexual commitment
Source: mBio. 2025 Aug 18;16(9):e00052-25. doi: 10.1128/mbio.00052-25 (PMC12421964; doi:10.1128/mbio.00052-25)
Supplement: Supplemental figures — Figures S1 to S4. [file mbio.00052-25-s0006.pdf]

Supplementary figures for

AP2X-1 is a negative regulator of *Toxoplasma gondii* sexual commitment

Li-Xiu Sun,[1,2] Meng Wang,[1] Tian-Yu Zhang,[1,2] Hany M Elsheikha,[3] Zhi-Wei Zhang,[1] Xiao-Nan Zheng,[4] Bao-Quan Fu,[1] Xing-Quan Zhu,[4] Guo-Hua Liu,[2] Jin-Lei Wang[1]

[1]State Key Laboratory for Animal Disease Control and Prevention, Key Laboratory of Veterinary Parasitology of Gansu Province, Lanzhou Veterinary Research Institute, Chinese Academy of Agricultural Sciences, Lanzhou, Gansu Province 730046, People's Republic of China

[2]Research Center for Parasites &Vectors, College of Veterinary Medicine, Hunan Agricultural University, Changsha, Hunan Province 410128, People's Republic of China

[3]School of Veterinary Medicine and Science, Faculty of Medicine and Health Sciences, University of Nottingham, Sutton Bonington Campus, Loughborough, LE12 5RD, UK.

[4]Laboratory of Parasitic Diseases, College of Veterinary Medicine, Shanxi Agricultural University, Taigu, Shanxi Province 030801, People's Republic of China

---

Address correspondence to Xing-Quan Zhu, xingquanzhu1@hotmail.com, Guo-Hua Liu, liuguohua5202008@163.com, Jin-Lei Wang, wangjinlei90@126.com.

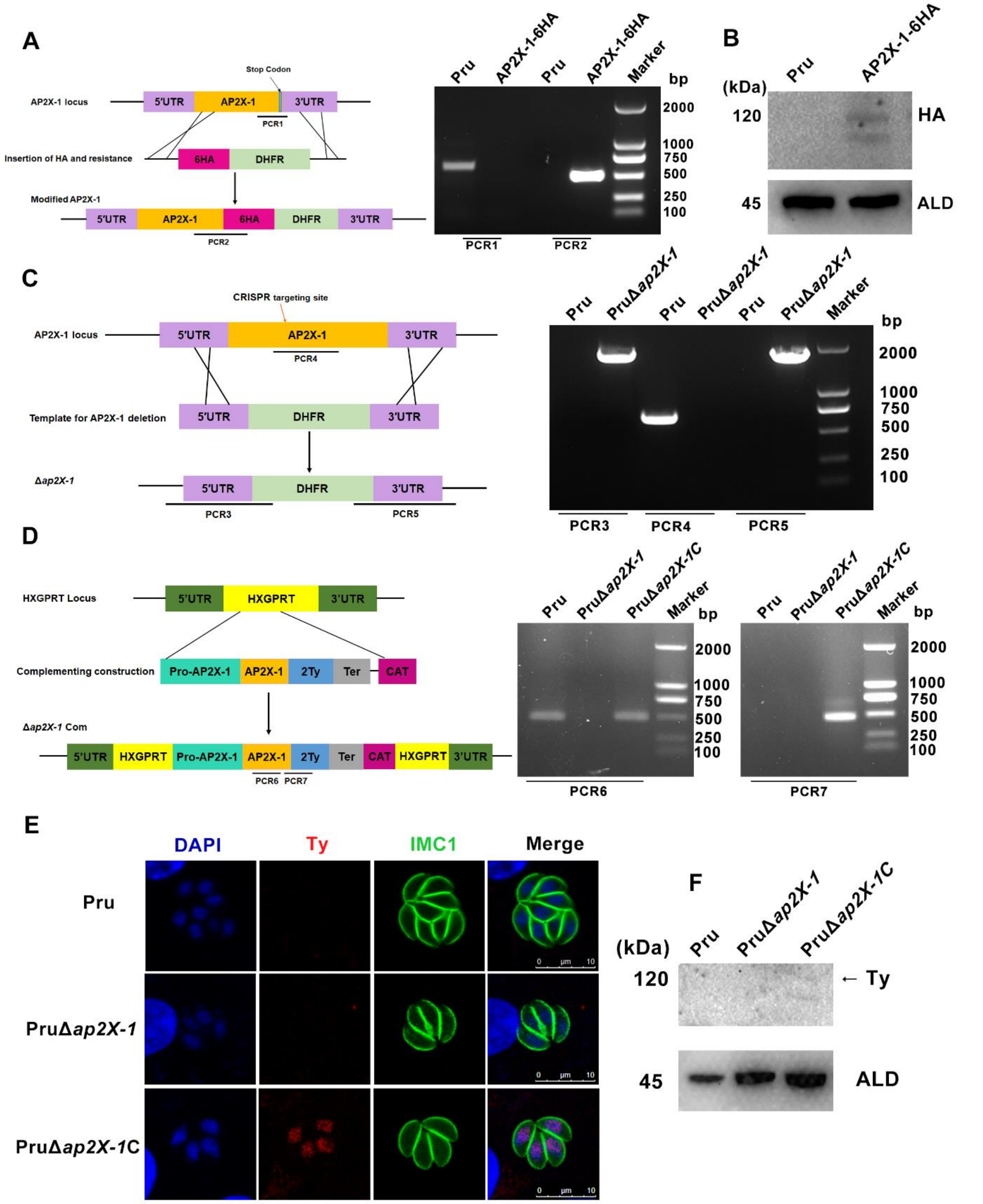

Supplementary FIG 1 Construction and validation of AP2X-1-6HA, PruΔ*ap2X-1* and complemented PruΔ*ap2X-1* (PruΔ*ap2X-1*C) strains. (A) Schematic illustration depicts the strategy used to generate the Pru::AP2X-1-6HA strain (referred to as AP2X-1-6HA) and confirms the strain's identity via diagnostic PCRs. PCR1 and PCR2 were designed to detect the successful insertion of C-terminal AP2X-1 by 6HA fragment. (B) Western blotting confirms the successful expression of AP2X-1-6HA in tachyzoites.

Parasite lysates were incubated with anti-HA antibody, with anti-aldolase (ALD) used as the loading control. (C) A schematic illustration shows the strategy used for deleting *ap2X-1* gene via CRISPR-Cas9 mediated gene homologous recombination, with diagnostic PCRs confirming the knockout strains. PCR3 and PCR5 verify the 5′ and 3′ integration of the selection marker, whereas PCR4 confirms the successful deletion of *ap2X-1* gene. (D) A schematic illustration shows the strategy used for complementation of *ap2X-1* with 2Ty-tagged driven by its own promoter at the hypoxanthine-xanthine-guanine phosphoribosyl transferase (HXGPRT) locus using CRISPR-Cas9 approach, along with diagnostic PCRs for PruΔ*ap2X-1*C. PCR6 detects the replacement of the HXGPRT locus by the coding sequence of *ap2X-1* and PCR7 verifies the insertion of C-terminal AP2X-1 by 2Ty fragment. (E) IFA demonstrates successful expression of AP2X-1-2Ty in PruΔ*ap2X-1*C strain. Colours indicate: green, anti-IMC1; red, anti-Ty; blue, DAPI. Scale bar, 10 μm. (F) Western blotting confirms expression of AP2X-1 in the PruΔ*ap2X-1*C strain. Parasite lysates were incubated with anti-Ty, with anti-aldolase (ALD) used as the loading control.

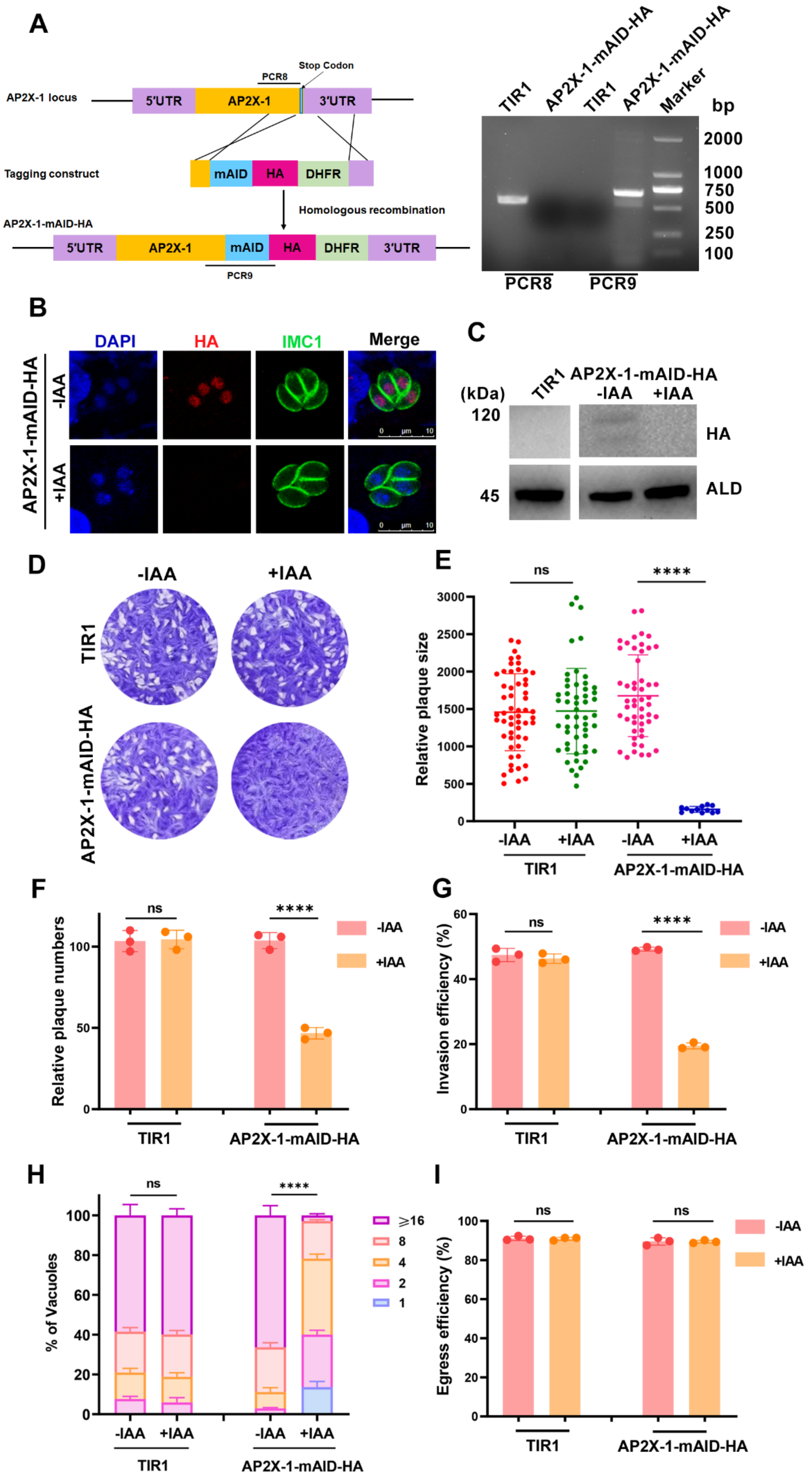

A
PCR8
Stop Codon
AP2X-1 locus
5'UTR
AP2X-1
3'UTR
Tagging construct
mAID
HA
DHFR
Homologous recombination
AP2X-1-mAID-HA
5'UTR
AP2X-1
mAID
HA
DHFR
3'UTR
PCR9
TIR1
AP2X-1-mAID-HA
TIR1
AP2X-1-mAID-HA
Marker
bp
2000
1000
750
500
250
100
PCR8
PCR9
B
DAPI
HA
IMC1
Merge
AP2X-1-mAID-HA
-IAA
+IAA
C
(kDa)
TIR1
AP2X-1-mAID-HA
-IAA
+IAA
120
HA
45
ALD
D
-IAA
+IAA
TIR1
AP2X-1-mAID-HA
E
ns
****
Relative plaque size
-IAA
+IAA
-IAA
+IAA
TIR1
AP2X-1-mAID-HA
F
Relative plaque numbers
ns
****
-IAA
+IAA
TIR1
AP2X-1-mAID-HA
G
Invasion efficiency (%)
ns
****
-IAA
+IAA
TIR1
AP2X-1-mAID-HA
H
% of Vacuoles
ns
****
≥16
8
4
2
1
-IAA
+IAA
-IAA
+IAA
TIR1
AP2X-1-mAID-HA
I
Egress efficiency (%)
ns
ns
-IAA
+IAA
TIR1
AP2X-1-mAID-HA

Supplementary FIG 2 Conditional depletion of AP2X-1 leads to growth defects of *T. gondii in vitro*. (A) A schematic illustration shows the strategy used for depletion of AP2X-1 by CRISPR-Cas9 mediated gene homologous recombination, along with diagnostic PCRs to confirm the depletion strain. PCR8 and PCR9 were designed to detect the successful insertion of C-terminal AP2X-1 by mAID-HA fragment. (B) AP2X-1-mAID-HA is localized to the nucleus in the tachyzoites and is efficiently depleted upon treatment with IAA (3-indoleacetic acid). Colours indicate: green, anti-IMC1; red, anti-Ty; blue, DAPI. Scale bar, 10 μm. (C) Western blotting verifies the degradation of the AP2X-1-mAID-HA protein after IAA treatment. AP2X-1-mAID-HA was detected with anti-HA antibody, and anti-aldolase (ALD) antibody served as the loading control. (D) Representative images of the plaques formed by AP2X-1-mAID-HA strain grown in HFF monolayers for 9 days with or without IAA treatment. (E) Relative size of the plaques detected in (D). Data represents the mean ± SD from three independent experiments. Statistical significance was tested by a two-tailed, unpaired *t*-test, $^{***}p < 0.0001$. (F) The number of plaques detected in (D). Data represents the mean ± SD for three independent experiments. Statistical analysis was performed by a two-tailed, unpaired *t*-test, $^{****}p < 0.0001$. (G) Invasion assay of the indicated strains grown in HFF monolayers with or without IAA treatment. Data represents the mean ± SD from three independent experiments. Statistical analysis was performed using a two-tailed, unpaired *t*-test, $^{****}p < 0.0001$. (H) Intracellular replication assay of the indicated strains grown in HFFs for 36 h with or without IAA treatment. At least 100 parasitophorous vacuoles (PVs) of each strain were examined in the experiment. Data represent the mean ± SD for three independent experiments, with statistical analysis performed by two-way ANOVA with Tukey tests for multiple comparisons, $^{****}p < 0.0001$. (I) Egress assay of the indicated strains with or without IAA treatment. At least 100 PVs of each strain were examined in the experiment. Data represents the mean ± SD for three independent experiments, with statistical analysis performed using a two-tailed, unpaired *t*-test, ns, not significant, $p > 0.05$.

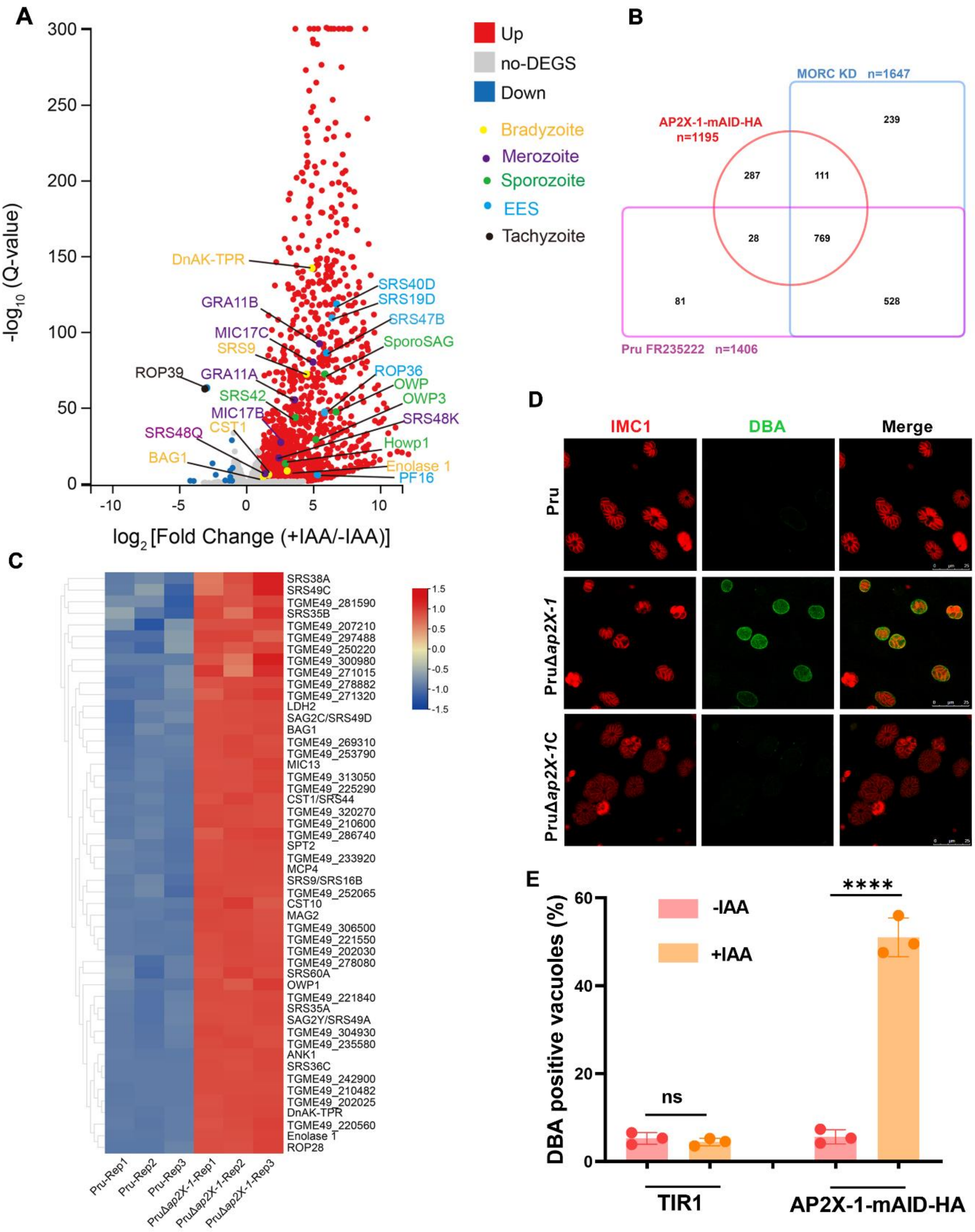

Supplementary FIG 3 Depletion of AP2X-1 induces the transcription of bradyzoite and sexual stage genes. (A) Volcano plot shows the dysregulated genes in AP2X-1-expressing and AP2X-1-depleted strains under normal culture conditions for 3 days. The merozoite, bradyzoite, sporozoite and tachyzoite highly expressed genes were analyzed from Supplementary dataset S3. Data from three biological replicates are plotted, with a fold change of ≥ 2.0 or ≤ -2.0 and $p < 0.05$ considered statistically significant. (B) Comparison of upregulated genes after depletion of AP2X-1, depletion of MORC, and FR235222-induced inhibition of HDAC3. (C) Heat map shows the upregulation of selected bradyzoite

highly expressed genes after knockout of *ap2X-1*. The color scale indicates log_2-transformed fold changes. (D) Representative IFA images of vacuoles formed by Pru, PruΔ*ap2X-1* and PruΔ*ap2X-1*C strains under normal culture conditions for 48 h. The vacuoles were stained with DBA (green), the parasites were stained with IMC1 (red). Scale bar, 25 μm. (E) Depletion of AP2X-1 increases the spontaneous conversion from tachyzoites to bradyzoites in normal medium with or without IAA. Data represent mean ± SD form three biological replicates, with the percentage of DBA positive PVs calculated from at least 100 PVs per replicate. Statistical analysis was performed using a two-tailed, unpaired *t*-test, $^{****}p < 0.0001$.

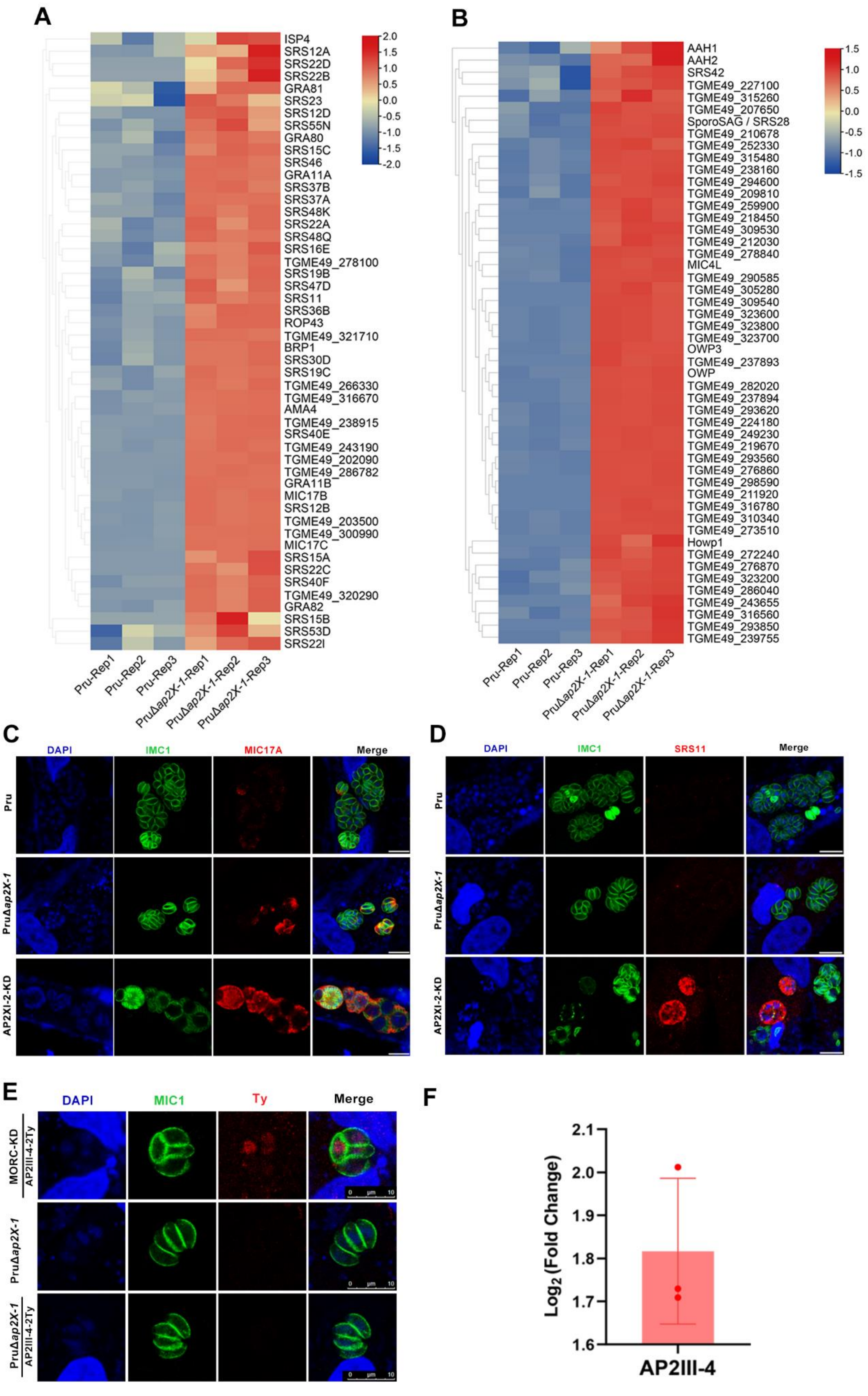
A
B
C
D
E
F
Pru-Rep1
Pru-Rep2
Pru-Rep3
PruΔap2X-1-Rep1
PruΔap2X-1-Rep2
PruΔap2X-1-Rep3
DAPI
IMC1
MIC17A
SRS11
MIC1
Ty
Merge
Pru
PruΔap2X-1
AP2XI-2-KD
MORC-KD
AP2III-4-2Ty
Log2(Fold Change)
AP2III-4

Supplementary FIG 4 Knockout of *ap2X-1* induces the transcription of sexual stage genes. (A) Heat map illustrates the upregulation of merozoite-associated genes following the knockout of *ap2X-1*. The color scale represents log_2-transformed fold changes. (B) Heat map shows the upregulation of sporozoite-associated genes after knockout of *ap2X-1.* The color scale represents log_2-transformed fold changes. (C) Knockout of *ap2X-1* activates the merozoite-specific protein MIC17A, as determined by IFA in PruΔ*ap2X-1* strain. Pru-AP2XI-2-mAID-6HA strain treated with IAA was used as a positive control. Colours indicate: green, anti-IMC1; red, anti-MIC17A; blue, DAPI. Scale bar, 10 µm. (D) The merozoite-specific protein SRS11 was nearly undetected in PruΔ*ap2X-1,* as shown by IFA. Pru-AP2XI-2-mAID-6HA strain treated with IAA was used as a positive control. Colours indicate: green, anti-IMC1; red, anti-SRS11; blue, DAPI. Scale bar, 10 µm. (E) AP2III-4 was detected in Pru-MORC-mAID-6HA strain treated with IAA but not undetected in PruΔ*ap2X-1* strain by IFA. Pru-MORC-mAID-6HA strain treated with IAA was used as a positive control. Colours indicate: green, anti-IMC1; red, anti-Ty; blue, DAPI. Scale bar, 10 µm. (F) Expression verification of the up-regulated AP2III-4 gene by real time quantitative PCR (RT-qPCR) in PruΔ*ap2X-1* strain. *β*-tubulin was used as a normalization control.
