# Supplementary material for: AP2X-1 is a negative regulator of Toxoplasma gondii sexual commitment
Source: mBio. 2025 Aug 18;16(9):e00052-25. doi: 10.1128/mbio.00052-25 (PMC12421964; doi:10.1128/mbio.00052-25)
Supplement: Table S1 — Primers used in the study. [file mbio.00052-25-s0007.docx]

**Supplementary Table S1 Primers used in the study.**

| Primer names | Primer sequence (5′-3′) | Use |
| --- | --- | --- |
| SgRNA-AP2X-1-Tag  SgRNA-AP2X-1-Tag-Fw  SgRNA-AP2X-1-Tag-Rv  AP2X-1-HR-Fw  AP2X-1-HR-Rv  PCR1-AP2X-1-Tag-Fw  PCR2-AP2X-1-Tag-Fw  PCR1-AP2X-1-Tag-Rv  PCR2-AP2X-1-Tag-Rv  SgRNA-AP2X-1-KO  SgRNA-AP2X-1-KO-Fw  SgRNA-AP2X-1-KO-Rv  U5-AP2X-1-Fw  U5-AP2X-1-Rv  U3-AP2X-1-Fw  U3-AP2X-1-Rv  DHFR-Fw  DHFR-Rv  pUC19-Fw  pUC19-Rv  PCR3-AP2X-1-Fw  PCR3-AP2X-1-Rv  PCR4-AP2X-1-Fw  PCR4-AP2X-1-Rv  PCR5-AP2X-1-F  PCR5-AP2X-1-R  AP2X-1-KZ-Fw  AP2X-1-KZ-Rv  Pro-AP2X-1-F  Pro-AP2X-1-R  AP2X-1-CDS-Fw  AP2X-1-CDS-Rw  HXGPRT-Pro-KZ-Fw  HXGPRT-Ter-KZ-Rv  SgRNA-HXGPRT  PCR6-AP2X-1-Tag-Fw  PCR7-AP2X-1-Tag-Fw  PCR6-AP2X-1-Tag-Rv  PCR7-AP2X-1-Tag-Rv  PCR8-AP2X-1-Tag-Fw  PCR9-AP2X-1-Tag-Fw  PCR8-AP2X-1-Tag-Rv  PCR9-AP2X-1-Tag-Rv  Sg-BFD1-Tag-Fw  BFD1-3-HR-F_W_  BFD1-3-HR-Rv  BFD1-Tag-Fw  SgRNA-AP2III-4-Tag-Fw  SgRNA-AP2III-4-Tag-Rv  AP2III-4-HR-Fw  AP2III-4-HR-Rv  AP2III-4-Tag-Fw  AP2III-4-Tag-Rv  RT-beta tubulin-Fw  RT-beta tubulin-Rv  RT-AP2III-4-Fw  RT-AP2III-4-Rv | GGGGCGAGGTAGTCCAGAAC  GGGGCGAGGTAGTCCAGAACGTTTTAGAGCTAGAAATAGC  AACTTGACATCCCCATTTAC GCGTCTGTCGCTTCGTCCACCCTGGGGGGCTTCGGGGCGAGGGCTAGCAAGGGCTCGGG  CCCTCGACTGTCAAGAGACGCCAGGCAACCGCCTCGCCTGTTATACGACTCACTATAGG  CTCGCCGCAGTCGCAGCAGAA  CTCGCCGCAGTCGCAGCAGAA  CACCCCACGCCCTTCCCTCAA  ATTATACCCGTGTGTTACG  GAGGACTCGCGAGTCCACGC  GAGGACTCGCGAGTCCACGCGTTTTAGAGCTAGAAATAGC  AACTTGACATCCCCATTTAC  GGTTTTCCCAGTCACGACGTTGGGAAAAGAAGAGGCGAGTGG  GGATTTACAGCCTGGCGAAGCTTAGACCTGCTGACGCGGAAGAC  CTATGCACTTGCAGGATGAATTCCACCTGAGAAAGTGACTGGGAGAA  GAGCGGATAACAATTTCACATCTGTGACTGTCTGGGCGTGTT  AAGCTTCGCCAGGCTGTAAATCC  GAATTCATCCTGCAAGTGCATAG  TGTGAAATTGTTATCCGCTC  AACGTCGTGACTGGGAAAACC  TTGTCGTGTGTGCGAGAAGCTCTG  GCCAAAGTAGAAAGGAATTAGCAT  AAGAAGAAGGCGGGGCAGGTC  ACCGCGTTCGCTTCCTTGTAT  TGACGCAGATGTGCGTGTATCCAC  GGCGTTTAGTCGGGAAGTTAACGT  GGGAAAAGAAGAGGCGAGTGG  TCTGTGACTGTCTGGGCGTGTT  AGTGGAGGACGGGAATTCGGGCCCCTTGCCTCTTTTAAAAGGCACAGT  ATGTGCCAAGAGAGAAAGCCGAGGGCTAGCAAGGGCTCGGG  ATGTGCCAAGAGAGAAAGCCGAGG  GACCTCGCCCGAGCCCTTGCTAGCCCTCGCCCCGAAACCCCCCAGGGT  GCTGATTTCGCCTTTCTGTGAGTTCATTCGTGCAGAGT  CTGTATCTTCAGCTTGCGAGGCAATTGACCCACCGTGT  GTTCATTCGTGCAGAGTACA  CTCGCCGCAGTCGCAGCAGAA  CTCGCCGCAGTCGCAGCAGAA  CACCCCACGCCCTTCCCTCAA  ATTATACCCGTGTGTTACG  CTCGCCGCAGTCGCAGCAGAA  CTCGCCGCAGTCGCAGCAGAA  CACCCCACGCCCTTCCCTCAA  ATTATACCCGTGTGTTACG  TTGATGTAACAGATGGAAGAGTTTTAGAGCTAGAAATAGC  TTACAAAACCGCCAGCCACCTTTGCATTCGGGGGGCTTGATGGCTAGCAAGGGCTCGGG  CCACGACTCTACACGGGGAGGAAGGACGTCAACAGACCCTCTATACGACTCACTATAGG  TTCAAGCCATATATCCATATGTG  GCTAGAGAAGCAACTGAGCAGTTTTAGAGCTAGAAATAGC  AACTTGACATCCCCATTTAC  GGCGAGAGCAGTGTTCCAGACTCCCTTGCTCAGTTGCTTCTCGCTAGCAAGGGCTCGGG  AAGTCAGCGCAAGCGAGTCTCTCGCGCTAGAGAAGCAACTGAATACGACTCACTATAGG  CAGACTCGTCTTCGGGTCATT  ATTATACCCGTGTGTTACG  GCAGCCATGAGCGGTGTGAC  GGAGACGAGGGAACGGGATGAG  GCAAGCGAGAGCGGCATCAG  GTGTGCGGGCGTCTTCAGTTC | SgRNA of the CRISPR plasmid for tagging AP2X-1 with 6HA  Construct the CRISPR plasmid for tagging AP2X-1 with 6HA  Construct the CRISPR plasmid for tagging AP2X-1 with 6HA  Amplify 6HA-DHFR fragment with the homologous arms of AP2X-1  Amplify 6HA-DHFR fragment with the homologous arms of AP2X-1  Detect the replacement of C-terminal AP2X-1 by 6HA fragment in PCR1  Detect the insert 6×HA fragment in PCR2  Detect the replacement of C-terminal AP2X-1 by 6HA fragment in PCR1  Detect the insert 6×HA fragment in PCR2  SgRNA of the CRISPR plasmid for deleting AP2X-1  Construct the CRISPR plasmid for deleting AP2X-1  Construct the CRISPR plasmid for deleting AP2X-1  Amplify the 5' homologous arms of AP2X-1 to construct the pUPRT-DHFR-D plasmid  Amplify the 5' homologous arms of AP2X-1 to construct the pUPRT-DHFR-D plasmid  Amplify the 3' homologous arms of AP2X-1 to construct the pUPRT-DHFR-D plasmid  Amplify the 3' homologous arms of AP2X-1 to construct the pUPRT-DHFR-D plasmid  Amplify the DHFR fragment to construct the pUPRT-DHFR-D plasmid  Amplify the DHFR fragment to construct the pUPRT-DHFR-D plasmid  Amplify the pUC19 fragment to construct the pUPRT-DHFR-D plasmid  Amplify the pUC19 fragment to construct the pUPRT-DHFR-D plasmid  Detect the insertion of 5' homologous fragment of AP2X-1 in PCR3  Detect the insertion of 5' homologous fragment of AP2X-1 in PCR3  Detect the deletion of AP2X-1 in PCR4  Detect the deletion of AP2X-1 in PCR4  Detect the insertion of 3' homologous fragment of AP2X-1 in PCR5  Detect the insertion of 3' homologous fragment of AP2X-1 in PCR5  Amplify the 5UTR-DHFR-3UTR fragment of AP2X-1  Amplify the 5UTR-DHFR-3UTR fragment of AP2X-1  Amplify the promoter of AP2X-1  Amplify the promoter of AP2X-1  Amplify the CDS of AP2X-1  Amplify the CDS of AP2X-1  Amplify the complementing fragment of AP2X-1 to construct Pru∆*ap2X-1*C strains  Amplify the complementing fragment of AP2X-1 to construct Pru∆*ap2X-1*C strains  SgRNA of CRISPR plasmid for AP2X-1 complement in HXGPRT locus  Detect the replacement of GPRT by *ap2X-1* of Pru∆*ap2X-1*C strain in PCR6  Detect the insertion of complementing fragment of Pru∆*ap2X-1*C strain in PCR7  Detect the replacement of GPRT by *ap2X-1* of Pru∆*ap2X-1*C strain in PCR6  Detect the insertion of complementing fragment of Pru∆*ap2X-1*C strain in PCR7  Detect the replacement of C-terminal AP2X-1 by mAID-HA fragment in PCR8  Detect the insertion of mAID-HA in PCR9  Detect the replacement of C-terminal AP2X-1 by mAID-HA fragment in PCR8  Detect the insertion of mAID-HA in PCR9  Construct the CRISPR plasmid for tagging BFD1 with 2Ty-HXGPRT  Amplify 2Ty-HXGPRT fragment with the homologous arms of BFD1  Amplify 2Ty-HXGPRT fragment with the homologous arms of BFD1  Detect the insert 2Ty-HXGPRT fragment of BFD1  Construct the CRISPR plasmid for tagging AP2III-4 with 2Ty-HXGPRT  Construct the CRISPR plasmid for tagging AP2III-4 with2Ty-HXGPRT  Amplify 2Ty-HXGPRT fragment with the homologous arms of AP2III-4  Amplify 2Ty-HXGPRT fragment with the homologous arms of AP2III-4  Detect the insert 2Ty-HXGPRT fragment of AP2III-4  Detect the insert 2Ty-HXGPRT fragment of AP2III-4  qRT-PCR of β-tubulin  qRT-PCR of β-tubulin  qRT-PCR of AP2III-4  qRT-PCR of AP2III-4 |
